# Supplementary material for: De novo diploid genome assembly using long noisy reads
Source: Nat Commun. 2024 Apr 5;15:2964. doi: 10.1038/s41467-024-47349-7 (PMC10997618; doi:10.1038/s41467-024-47349-7)
Supplement: Supplementary file 1 — Supplementary Information [file 41467_2024_47349_MOESM1_ESM.pdf]

Supplementary Information for

de novo diploid assembly using long noisy reads

Nie *et al.*

Supplementary Note 1: The details of the datasets

The dataset of *S. cerevisiae* (SK×Y12) is a pseudo-diploid dataset combining two haploid yeast strains SK1 and Y12. For each yeast strain, we downsampled 100X data. For *D. melanogaster* (ISO1×A4), we used the same data as Sergey's paper[1] for a fair comparison. The dataset is available at https://obj.umiacs.umd.edu/marbl_publications/hicanu/index.html. *A. thaliana* (Col-0 × C24) was generated using our in-house sequencing.

A. thaliana (Col-0 × C24)

Plant materials and growth conditions

The Arabidopsis Columbia-0 (Col-0) and C24 ecotypes were used as the parental lines, and the F1 hybrids between Col-0 and C24 were generated[2]. Seeds were surface sterilized and then grown on Murashige and Skoog (MS) medium agar plates (½ MS salts, 2% sucrose, 1.2% agar, pH 5.7) for 7 days. The 7d-old seedlings were transplanted on soil pots and grown for three weeks at 22 ± 1°C with a 16-h-light/8-h-dark photoperiod. The whole seedlings were then harvested for subsequent analysis. The authenticity of F1 progenies from Col and C24 hybridization was genotyped[2].

DNA extraction and purification

Samples were collected, and high molecular weight genomic DNA was prepared by the CTAB method and followed by purification with a QIAGEN® Genomic kit (Cat#13343, QIAGEN) for regular sequencing, according to the standard operating procedure provided by the manufacturer. Ultra-long DNA was extracted by the SDS method without a purification step to sustain the length of DNA. The DNA degradation and contamination of the extracted DNA were monitored on 1% agarose gels. DNA purity was then detected using NanoDrop™ One UV-Vis spectrophotometer (Thermo Fisher Scientific, USA), of which OD260/280 ranges from 1.8 to 2.0 OD 260/230 is

between 2.0-2.2. At last, DNA concentration was further measured by Qubit® 4.0 Fluorometer (Invitrogen, USA).

Nanopore whole genome sequencing and base-calling

A total amount of 3-4 μg DNA per sample was used as input material for the ONT library preparations. After the sample was qualified, size-select of long DNA fragments was performed using the PippinHT system (Sage Science, USA). Next, the ends of DNA fragments were repaired, and A-ligation reactions were conducted with NEBNext Ultra II End Repair/dA-tailing Kit (Cat# E7546). The adapter in the SQK-LSK109 (Oxford Nanopore Technologies, UK) was used for further ligation reaction and the DNA library was measured by Qubit® 4.0 Fluorometer (Invitrogen, USA). About 700ng DNA library was constructed and performed on a Nanopore PromethION sequencer instrument (Oxford Nanopore Technologies, UK) at the Genome Center of Grandomics (Wuhan, China). The raw data, collected in this experiment, were obtained as fast5 files after the conversion of electrical signals into base calls via Guppy v5.0.16 (Oxford Nanopore Technologies).

Simulated data

We first generated paternal and maternal haplotype genomes based on the chromosome chrIV of the yeast SK1 genome. We ran the command to randomly change the bases so that the heterozygosity rate of the dataset was 0.01, 0.005, 0.001, 0.0005, and 0.0001 respectively.

```
Python3 $PECAT/scripts/fxtool.py fx_gen_dipref -r sk1.fa -c chrIV -f fa_sk1_chrIV.fa -m mo_sk1_chrIV.fa -s variants_sites.txt -p 0.01
```

Next, we ran pbsim2 (eeb5a19)[3] to simulate PacBio CLR reads and Nanopore reads with the following commands.

```
pbsim --depth 30 --prefix fa --hmm_model $PBSIM2/data/P6C4.model fa_sk1_chrIV.fa --length-mean 25000 --id-prefix F
```

```
pbsim --depth 30 --prefix mo --hmm_model $PBSIM2/data/P6C4.model mo_sk1_chrIV.fa --length-mean 25000 --id-prefix M
```

```
pbsim --depth 30 --prefix fa --hmm_model $PBSIM2/data/R103.model fa_sk1_chrIV.fa --length-mean 25000 --id-prefix F
```

```
pbsim --depth 30 --prefix mo --hmm_model $PBSIM2/data/R103.model mo_sk1_chrIV.fa --length-mean 25000 --id-prefix M
```

Supplementary Note 2: Commands for error correction and assembly

Commands for Canu and Purge_dups

Canu (v2.1)[4] was run with the following commands for PacBio CLR reads and Nanopore reads:

```
canu -p $SPECIES -d $SPECIES genomeSize=$GENOME_SIZE corOutCoverage=100 -correct -(pacbio|nanopore) $READS maxThreads=$THREADS
canu -p $SPECIES -d $SPECIES genomeSize=$GENOME_SIZE -corrected -(pacbio|nanopore) ./$SPECIES/$SPECIES.correctedReads.fasta.gz batOptions="-dg 6 -db 6 -dr 1 -ca 500 -cp 50" maxThreads=$THREADS
```

Purge_dups (v1.2.5)[5] was run with the following commands to partition the contigs generated by other assemblers to primary and alternate contigs.

```
minimap2 -x map-pb $CONTIGS $READS | gzip -c - > rd2ctg.paf.gz
pbcstat rd2ctg.paf.gz
calcuts PB.stat > cutoffs 2>calcults.log
split_fa $CONTIGS > contigs.split.fasta
minimap2 -xasm5 -DP contigs.split.fasta contigs.split.fasta | gzip -c - > ctg2ctg.paf.gz
purge_dups -2 -T cutoffs -c PB.base.cov ctg2ctg.paf.gz > dups.bed 2> purge_dups.log
get_seqs dups.bed $CONTIGS
```

Commands for FALCON and FALCON-Unzip

FALCON (1.8.1)[6] was run with the following parameters to assemble reads and FACON-Unzip (1.3.7)[6] was run with default parameters.

Parameters for *S. cerevisiae* (SK1×Y12):

```
genome_size=12000000
length_cutoff = 4000
length_cutoff_pr = 4000
pa_daligner_option = -e0.75 -l1800 -k18 -h240 -w8 -s100
ovlp_daligner_option = -k24 -h60 -e.96 -l1500 -s100
pa_HPCdaligner_option = -v -B128 -M24
ovlp_HPCdaligner_option = -v -B128 -M24
pa_DBsplit_option = -a -x500 -s50
ovlp_DBsplit_option = -s50
falcon_sense_option = --output_multi --min_idt 0.70 --min_cov 4 --
max_n_read 400 --n_core 24
overlap_filtering_setting = --max_diff 120 --max_cov 150 --min_cov 4
--n_core 2
falcon_sense_skip_contained = False
```

Parameters for *A. thaliana* (Col-0×Cvi-0):

```
genome_size=130000000
length_cutoff = 4000
length_cutoff_pr = 4000
pa_daligner_option = -e0.75 -l1800 -k18 -h240 -w8 -s100
ovlp_daligner_option = -k24 -h750 -e.96 -l1500 -s100
pa_HPCdaligner_option = -v -B128 -M24
ovlp_HPCdaligner_option = -v -B128 -M24
pa_DBsplit_option = -a -x500 -s400
ovlp_DBsplit_option = -s400
```

```
falcon_sense_option = --output_multi --min_idt 0.70 --min_cov 4 --max_n_read 400 --n_core 24
overlap_filtering_setting = --max_diff 80 --max_cov 120 --min_cov 4 --n_core 24
falcon_sense_skip_contained = False
```

Parameters for *D. melanogaster* (ISO1×A4):

```
genome_size=140000000
length_cutoff = 10000
length_cutoff_pr = 10000
pa_daligner_option = -e0.75 -l1200 -k18 -h480 -w8 -s100
ovlp_daligner_option = -k24 -h480 -e.95 -l1500 -s100
pa_HPCdaligner_option = -v -B128 -M24
ovlp_HPCdaligner_option = -v -B128 -M24
pa_DBsplit_option = -x500 -s400
ovlp_DBsplit_option = -s400
falcon_sense_option = --output_multi --min_idt 0.70 --min_cov 4 --max_n_read 200 --n_core 24
overlap_filtering_setting = --max_diff 1000 --max_cov 1000 --min_cov 2 --n_core 24
falcon_sense_skip_contained = False
```

Commands for MECAT2

MECAT2 (f54c542)[7] was run with the command “mecat.pl correct cfg” for correcting PacBio CLR reads. The parameter file is shown below:

```
PROJECT=$SPECIES
RAWREADS= $READS
GENOME_SIZE= $GENOME_SIZE
THREADS=THREADS
MIN_READ_LENGTH=2000
```

```
CNS_OVLP_OPTIONS="-kmer_size 13"
CNS_PCAN_OPTIONS="-p 100000 -k 100"
CNS_OPTIONS=""
CNS_OUTPUT_COVERAGE=80
TRIM_OVLP_OPTIONS="-skip_overhang"
TRIM_PM4_OPTIONS="-p 100000 -k 100"
TRIM_LCR_OPTIONS=""
TRIM_SR_OPTIONS=""
ASM_OVLP_OPTIONS=""
FSA_OL_FILTER_OPTIONS="--max_overhang=-1 --min_identity=-1"
FSA_ASSEMBLE_OPTIONS=""
```

To speed up correcting *B.taurus* (Angus × Brahman) reads, we adjusted the parameters as shown below:

```
CNS_OVLP_OPTIONS="-kmer_size 17"
```

Commands for NECAT

NECAT (47c6c23)[8] was run with the command "necat.pl correct cfg' for correcting Nanopore reads. The parameter file is shown below:

```
PROJECT= $SPECIES
ONT_READ_LIST=$READS
GENOME_SIZE=$GENOME_SIZE
THREADS=$THREADS
MIN_READ_LENGTH=3000
PREP_OUTPUT_COVERAGE=80
OVLP_FAST_OPTIONS=-n 500 -z 20 -b 2000 -e 0.5 -j 0 -u 1 -a 1000
OVLP_SENSITIVE_OPTIONS=-n 500 -z 10 -e 0.5 -j 0 -u 1 -a 1000
CNS_FAST_OPTIONS=-a 2000 -x 4 -y 12 -l 1000 -e 0.5 -p 0.8 -u 0
CNS_SENSITIVE_OPTIONS=-a 2000 -x 4 -y 12 -l 1000 -e 0.5 -p 0.8 -u 0
TRIM_OVLP_OPTIONS=-n 100 -z 10 -b 2000 -e 0.5 -j 1 -u 1 -a 400
```

```
ASM_OVLP_OPTIONS=-n 100 -z 10 -b 2000 -e 0.5 -j 1 -u 0 -a 400
NUM_ITER=2
CNS_OUTPUT_COVERAGE=80
CLEANUP=1
USE_GRID=false
GRID_NODE=0
GRID_OPTIONS=
SMALL_MEMORY=0
FSA_OL_FILTER_OPTIONS=
FSA_ASSEMBLE_OPTIONS=
FSA_CTG_BRIDGE_OPTIONS=
POLISH_CONTIGS=true
```

Commands for Flye + HapDup

Flye (2.9)[9] was run to assemble reads: *A. thaliana* (Col-0×C24), *B. taurus* (Bison × Simmental), and HG002. Flye was run with the command:

```
flye --nano-raw|--nano-hq|--pacbio-hifi $READS --out-dir $SPECIES --threads 48 --genome-size $GENOME_SIZE
```

For *B. taurus* (Bison × Simmental) and HG002, we added parameter `--asm-coverage 50`.

Then, HapDup (0.5 or 0.12)[10,11]was run with the command:

```
minimap2 assembly.fasta $READS -a -x map-ont  -t 48 | samtools sort -@4 -m 4G  > rd2ctg.sorted.bam

samtools index -@ 48 rd2ctg.sorted.bam

HD_DIR=`pwd`

singularity exec --bind $HD_DIR hapdup_0.5.sif hapdup --assembly $HD_DIR/assembly.fasta  --bam  $HD_DIR/rd2ctg.sorted.bam  --out-dir $HD_DIR/hapdup -t 48 --rtype ont
```

where `assembly.fasta` is the assembly generated by Flye. HapDup (0.12) was run assemble Nanopore R10 sequencing (ultra-long), Nanopore R10 duplex sequencing and PacBio HiFi sequencing reads.

Commands for Shasta

Shasta (0.9.0 or 0.11.1)[12] was run with the following command for Nanopore reads *A. thaliana* (Col-0×C24), *B. taurus* (Bison × Simmental), and HG002.

```
shasta-Linux-0.9.0 --input $READS --config (Nanopore-Phased-Jan2022|
Nanopore-UL-Phased-Jan2022) --threads $THREADS
```

Shasta (v0.11.1) was run to assemble Nanopore R10 sequencing (ultra-long) and Nanopore R10 duplex sequencing reads. Then, the following command was used to connect the haplotigs in "Assembly-Phased.fasta" file to generate primary/alternate-style contigs.

```
python3 $PECAT/fxtools.py fx_split_shasta Assembly-Phased.fasta
primary.fasta alternate.fasta --min-length 500
```

Commands for PECAT

PECAT (v0.0.2 or v0.0.3) was run to assemble genomes. PECAT (v0.0.3) was run to assemble Nanopore R10 sequencing (ultra-long), Nanopore R10 duplex sequencing and PacBio HiFi sequencing reads. PECAT was run with the command 'pecat.pl correct cfg' for correcting and the command 'pecat.pl unzip cfg' for assembling.

Parameters for *S. cerevisiae* (SK1×Y12):

```
project=yeast
reads= $READS
genome_size=12000000
threads=48
cleanup=1
grid=local

prep_min_length=3000
prep_output_coverage=80

corr_iterate_number=1
corr_block_size=4000000000
```

```
corr_filter_options=--
filter0=al=2000:alr=0.5:aalr=0.5:oh=1000:ohr=0.1
corr_correct_options=--score=weight:lc=10  --aligner  diff  --filter1
oh=1000
corr_rd2rd_options=-x ava-pb
corr_output_coverage=80

align_block_size=4000000000
align_rd2rd_options=-X -g3000 -w30 -k19 -m100 -r500
align_filter_options=--
filter0=l=5000:al=5000:aal=6000:aalr=0.5:oh=1000:ohr=0.1          --
task=extend --filter1=oh=100:ohr=0.01

asm1_assemble_options=--reducer1 spur:length=1000:nodesize=3

phase_method=0
phase_rd2ctg_options=-x map-pb -c -p 0.5 -r 1000
phase_use_reads=1
phase_phase_options= --phase_options icr=0.2
phase_filter_options=

asm2_assemble_options=--reducer1    spur:length=1000:nodesize=3    --
contig_format dual,prialt

polish_use_reads=1
polish_map_options = -x asm20
polish_cns_options =
```

A. thaliana (Col-0×Cvi-0) and *D. melanogaster* (ISO1×A4)

```
project= $SPCIES
reads= $READS
genome_size=$GENOME_SIZE
threads=48
cleanup=1
grid=local

prep_min_length=3000
prep_output_coverage=80

corr_iterate_number=1
corr_block_size=4000000000
corr_filter_options=--
filter0=:al=5000:alr=0.5:aal=8000:aalr=0.5:oh=2000:ohr=0.2
corr_correct_options=--score=weight:lc=10  --aligner  diff  --filter1
oh=1000:ohr=0.01
```

```
corr_rd2rd_options=-x ava-pb
corr_output_coverage=80

align_block_size=4000000000
align_rd2rd_options=-X -g3000 -w30 -k19 -m100 -r500
align_filter_options=--
filter0=l=3000:al=3000:alr=0.5:aalr=0.5:oh=1000:ohr=0.1 --task=extend
--filter1=oh=100:ohr=0.01

asm1_assemble_options=--max_trivial_length 10000

phase_rd2ctg_options=-x map-pb -c -p 0.5 -r 1000
phase_use_reads=1
phase_phase_options= --phase_options icr=0.2
phase_filter_options= --threshold=1000

asm2_assemble_options=--max_trivial_length    10000    --contig_format
dual,prialt

polish_use_reads=1
polish_map_options = -x asm20
polish_cns_options =
```

Parameters for *B. taurus* (Angus×Brahman):

```
project=cattle
reads= $READS
genome_size= 2700000000
threads=48
grid=local
cleanup=1

prep_min_length=3000
prep_output_coverage=80

corr_iterate_number=1
corr_block_size=8000000000
corr_filter_options=--
filter0=:al=5000:alr=0.5:aal=8000:aalr=0.5:oh=2000:ohr=0.2
corr_correct_options=--score=weight:lc=10 --aligner diff --filter1
oh=1000:ohr=0.01 --candidate n=300:f=20
corr_rd2rd_options=-x ava-pb -f 0.005 -I 20G
corr_output_coverage=80

align_block_size=4000000000
align_rd2rd_options=-X -g3000 -w30 -k19 -m100 -r500 -I 20G -f 0.005
```

```
align_filter_options=--
filter0=l=3000:al=3000:alr=0.5:aalr=0.5:oh=1000:ohr=0.1 --task=extend
--filter1=oh=100:ohr=0.01

asm1_assemble_options= --max_trivial_length 10000

phase_rd2ctg_options=-x map-pb -c -p 0.5 -r 1000
phase_use_reads=1
phase_phase_options= --phase_options icr=0.2
phase_filter_options= --threshold=1000

asm2_assemble_options= --max_trivial_length 10000 --contig_format
dual,prialt

polish_use_reads=1
polish_map_options = -x asm20
polish_filter_options = --filter0 oh=1000:ohr=0.1
polish_cns_options =
```

Parameters for *A. thaliana* (Col-0 × C24):

```
project= arab
reads= $READS
genome_size= 130000000
threads=48
cleanup=1
grid=local

prep_min_length=3000
prep_output_coverage=80

corr_iterate_number=1
corr_block_size=4000000000
corr_filter_options=--
filter0=l=5000:al=2500:alr=0.5:aal=5000:oh=3000:ohr=0.3
corr_correct_options=--score=weight:lc=10 --aligner edlib --filter1
oh=1000:ohr=0.01
corr_rd2rd_options=-x ava-ont -k19
corr_output_coverage=80

align_block_size=4000000000
align_rd2rd_options=-X -g3000 -w30 -k19 -m100 -r500 -f 0.001
align_filter_options=--
filter0=l=5000:aal=6000:aalr=0.5:oh=3000:ohr=0.3 --task=extend --
filter1=oh=300:ohr=0.03
```

```
asm1_assemble_options=--max_trivial_length 10000

phase_method=2
phase_rd2ctg_options=-x map-ont -c -p 0.5 -r 1000
phase_phase_options= --coverage lc=30 --phase_options
icr=0.1:icc=8:sc=10
phase_use_reads=1
phase_filter_options= --threshold=1000

phase_clair3_command = singularity exec -B `pwd -P`:`pwd -P`
clair3_v0.1-r12.sif /opt/bin/run_clair3.sh
phase_clair3_options=--platform=ont --
model_path=/opt/models/ont_guppy5/  --include_all_ctgs
phase_clair3_rd2ctg_options=-x map-ont -c -p 0.5 -r 1000
phase_clair3_phase_options= --coverage lc=30 --phase_options
icr=0.1:icc=6:sc=10 --filter i=70
phase_clair3_use_reads=0
phase_clair3_filter_options= --threshold=2500 --rate 0.05

asm2_assemble_options=--max_trivial_length 10000 --contig_format
dual,prialt

polish_map_options = -x map-ont -k19 -w10 -I 10g
polish_use_reads=0
polish_cns_options =

polish_medaka = 1
polish_medaka_command =singularity exec -B `pwd -P`:`pwd -P`
medaka_v1.7.2.sif medaka
polish_medaka_map_options = -x map-ont  -k19 -w10 -I 10g
polish_medaka_cns_options = --model r941_prom_sup_g507
```

Parameters for *B. taurus* (Bison × Simmental):

```
project= cattle
reads= $READS
genome_size= 2700000000
threads=48
cleanup=1
grid=local
prep_min_length=3000
prep_output_coverage=80
corr_iterate_number=1
corr_block_size=8000000000
corr_filter_options=--
filter0=l=5000:al=2500:alr=0.5:aal=8000:oh=3000:ohr=0.3
```

```
corr_correct_options=--score=weight:lc=16 --aligner edlib --filter1 
oh=1000:ohr=0.01 --candidate n=400:f=20
corr_rd2rd_options=-x ava-ont -f 0.005 -I 10G
corr_output_coverage=80
align_block_size=12000000000
align_rd2rd_options=-X -g3000 -w30 -k19 -m100 -r500 -I 10G -f 0.005
align_filter_options=--
filter0=l=5000:aal=6000:aalr=0.5:oh=3000:ohr=0.3 --task=extend --
filter1=oh=300:ohr=0.03
asm1_assemble_options=--max_trivial_length 1000000

phase_method=2
phase_rd2ctg_options=-x map-ont -c -p 0.5 -r 1000
phase_use_reads=1
phase_phase_options= --coverage lc=30 --phase_options 
icr=0.1:icc=8:sc=10
phase_filter_options = --threshold 1000

phase_clair3_command = singularity exec --containall -B `pwd -P`:`pwd 
-P` clair3_v0.1-r12.sif /opt/bin/run_clair3.sh
phase_clair3_options=--platform=ont --
model_path=/opt/models/ont_guppy5/  --include_all_ctgs
phase_clair3_rd2ctg_options=-x map-ont -c -p 0.5 -r 1000
phase_clair3_use_reads=0
phase_clair3_phase_options= --coverage lc=30 --phase_options 
icr=0.1:icc=6:sc=10 --filter i=70
phase_clair3_filter_options = --threshold 2500 --rate 0.05

asm2_assemble_options=--max_trivial_length 1000000 --contig_format 
dual,prialt
polish_map_options = -x map-ont -w10 -k19
polish_use_reads=0
polish_filter_options=--filter0 oh=2000:ohr=0.2:aalr=0.5
polish_cns_options =

polish_medaka = 1
polish_medaka_command = singularity exec -B `pwd -P`:`pwd -P` 
medaka_v1.7.2.sif medaka
polish_medaka_map_options = -x map-ont -w10 -k19
polish_medaka_cns_options = --model r941_prom_sup_g507
```

Parameters for HG002 (ONT R9 UL):

```
project= human
reads= $READS
genome_size= 3000000000
```

```
threads=48
cleanup=1
grid=local
prep_min_length=3000
prep_output_coverage=80
corr_iterate_number=1
corr_block_size=4000000000
corr_filter_options=--
filter0=l=5000:al=2500:alr=0.5:aal=8000:oh=3000:ohr=0.3
corr_correct_options=--score=weight:lc=10 --aligner edlib --filter1
oh=1000:ohr=0.01 --candidate n=600:f=30
corr_rd2rd_options=-x ava-ont -f 0.005 -I 10G
corr_output_coverage=80

align_block_size=12000000000
align_rd2rd_options=-X -g3000 -w30 -k19 -m100 -r500 -I 10G -f 0.002
align_filter_options=--
filter0=l=5000:aal=6000:aalr=0.5:oh=3000:ohr=0.3 --task=extend --
filter1=oh=300:ohr=0.03
asm1_assemble_options=--max_trivial_length 10000

phase_method=2
phase_rd2ctg_options=-x map-ont -c -p 0.5 -r 1000
phase_use_reads=1
phase_phase_options= --coverage lc=30 --phase_options
icr=0.1:icc=8:sc=10
phase_filter_options = --threshold 1000

phase_clair3_use_reads=0
phase_clair3_command = singularity exec -B `pwd -P`:`pwd -P`
clair3_v0.1-r12.sif /opt/bin/run_clair3.sh
phase_clair3_options=--platform=ont --
model_path=/opt/models/ont_guppy5/  --include_all_ctgs
phase_clair3_rd2ctg_options=-x map-ont -c -p 0.5 -r 1000
phase_clair3_phase_options= --coverage lc=30 --phase_options
icr=0.1:icc=3:sc=10 --filter i=70
phase_clair3_filter_options = --threshold 2500 --rate 0.05

asm2_assemble_options=--reducer0 "best:cmp=2,0.1,0.1|phase:sc=3" --
contig_format dual,prialt --min_identity 0.98

polish_map_options = -x map-ont -w10 -k19 -I 10g
polish_use_reads=0
polish_filter_options=--filter0 oh=2000:ohr=0.2:aalr=0.5
```

```
polish_cns_options =

polish_medaka = 1
polish_medaka_command = singularity exec -B `pwd -P`:`pwd -P` 
medaka_v1.7.2.sif medaka
polish_medaka_map_options = -x map-ont -w10 -k19
polish_medaka_cns_options = --model r941_prom_sup_g507
```

Parameters for HG002 (ONT R10 UL):

```
project= human
reads= $READS
genome_size= $GENOME_SIZE
threads=48
cleanup=0
compress=0
grid=auto
prep_min_length=3000
prep_output_coverage=80
corr_iterate_number=1
corr_block_size=4000000000
corr_filter_options=--
filter0=l=5000:al=2500:alr=0.5:aal=8000:oh=3000:ohr=0.3
corr_correct_options=--score=weight:lc=10 --aligner 
edlib:bs=1000:mc=6  --min_coverage 4 --filter1 oh=1000:ohr=0.01 --
candidate n=600:f=30 --min_identity 90 --min_local_identity 80
corr_rd2rd_options=-x ava-ont -f 0.005 -I 10G
corr_output_coverage=60
align_block_size=12000000000
align_rd2rd_options=-X -g3000 -w30 -k19 -m100 -r500 -I 10G -f 0.002
align_filter_options=--
filter0=l=5000:aal=6000:aalr=0.5:oh=3000:ohr=0.3 --task=extend --
filter1=oh=300:ohr=0.03 --min_identity 0.95
asm1_assemble_options=--max_trivial_length 10000

phase_method=2
phase_rd2ctg_options=-x map-ont -w 10 -k19  -c -p 0.5 -r 1000 -I 10G 
-K 8G
phase_use_reads=1
phase_phase_options= --coverage lc=20 --phase_options 
icr=0.1:icc=8:sc=10
phase_filter_options = --threshold 1000

phase_clair3_command = singularity exec --containall -B `pwd -P`:`pwd 
-P` -B /tmp:/tmp clair3_v0.1-r12.sif /opt/bin/run_clair3.sh
```

```
phase_clair3_rd2ctg_options=-x map-ont -w10 -k19 -c -p 0.5 -r 1000 -I
10G -K 8G
phase_clair3_use_reads=0
phase_clair3_phase_options= --coverage lc=20 --phase_options
icr=0.1:icc=3:sc=10 --filter i=90
phase_clair3_filter_options = --threshold 2500 --rate 0.05
phase_clair3_options=--platform=ont --
model_path=/opt/models/r941_prom_sup_g5014  --include_all_ctgs

asm2_assemble_options=--reducer0 "best:cmp=2,0.1,0.1|phase:sc=3" --
contig_format prialt,dual

polish_map_options = -x map-ont -w10 -k19 -I 10g -K 8G -a
polish_cns_options =
polish_use_reads=0
polish_filter_options=--filter0 oh=2000:ohr=0.2:i=96

polish_medaka = 1
polish_medaka_command = singularity exec --containall -B `pwd -
P`:`pwd -P` medaka_v1.7.2.sif medaka
polish_medaka_map_options = -x map-ont -w10 -k19 -I 10g -K 8G
polish_medaka_cns_options = --model r1041_e82_260bps_sup_g632
polish_medaka_filter_options=--filter0 oh=2000:ohr=0.2:i=96
```

Parameters for HG002 (ONT R10 Duplex):

```
project= human
reads= $READS
genome_size= $GNOME_SIZE
threads=40
cleanup=0
grid=auto
prep_min_length=3000
prep_output_coverage=60
corr_iterate_number=1
corr_block_size=4000000000
corr_correct_options=--score=weight:lc=8 --aligner diff:s=500 --
min_coverage 1 --filter1 oh=1000:ohr=0.01 --min_identity 95 --
min_local_identity 90 --candidate n=600:f=30
corr_filter_options=--
filter0=l=5000:al=2500:alr=0.5:aal=5000:oh=3000:ohr=0.3
corr_rd2rd_options=-X -g3000 -w30 -k19 -m100 -r500 -f 0.002 -K8G -I
8G
corr_output_coverage=60
align_block_size=4000000000
```

```
align_rd2rd_options=-X -g3000 -w30 -k19 -m100 -r500 -f 0.002 -K8G -I 
8G
align_filter_options=--
filter0=l=5000:aal=6000:aalr=0.5:oh=3000:ohr=0.3 --task=extend --
filter1=oh=50:ohr=0.01  --aligner diff:s=100 --min_identity 0.90
asm1_assemble_options= --min_identity 0.99 --min_coverage 1

phase_method=2
phase_rd2ctg_options=-x map-ont -w10 -k19 -c -p 0.5 -r 1000 -I 10G
phase_use_reads=1
phase_phase_options= --coverage lc=8 --phase_options 
icr=0.02:icc=3:sc=4 --
filter=i=95.00:alr=0.80:oh=100:ohr=0.01:ilid=100

phase_clair3_command=singularity exec --containall -B `pwd -P`:`pwd -
P` -B /tmp:/tmp clair3_v0.1-r12.sif /opt/bin/run_clair3.sh
phase_clair3_use_reads=0
phase_clair3_options=--platform=ont --
model_path=/opt/models/ont_guppy5/  --include_all_ctgs
phase_clair3_rd2ctg_options=-x map-ont -w10 -k19 -c -p 0.5 -r 1000 -I 
10G -K 8G
phase_clair3_phase_options=--coverage lc=8 --phase_options 
icr=0.02:icc=2:sc=4 --filter i=95
phase_clair3_filter_options=--threshold=2500 --rate 0.05

asm2_assemble_options= --reducer0 "best:cmp=2,0.1,0.1|phase:sc=2" --
min_identity 0.99 --max_trivial_length 10000 --contig_format 
dual,prialt --min_coverage 1

polish_map_options=-x map-ont -w10 -k19 -I 10G -K 8G -a
polish_filter_options=--filter0 oh=1000:ohr=0.1:i=98
polish_cns_options=
polish_medaka=1
polish_medaka_command= singularity exec --containall -B `pwd -P`:`pwd 
-P` medaka_v1.7.2.sif medaka
polish_medaka_map_options=-x map-ont -w10 -k19 -I 10G -K 8G
polish_medaka_cns_options = --model r1041_e82_400bps_sup_g615
polish_medaka_filter_options=--filter0 oh=1000:ohr=0.1:i=98
```

Parameters for HG002 (PacBio HiFi):

```
project= human
reads= $READS
genome_size=$GENOME_SIZE
threads=48
cleanup=0
```

```
grid=auto
prep_min_length=3000
prep_output_coverage=60
corr_iterate_number=1
corr_block_size=4000000000
corr_correct_options=--score=weight:lc=8 --aligner diff:s=100 --
min_coverage 1 --filter1 oh=100 --min_identity 96 --
min_local_identity 95
corr_filter_options=--
filter0=l=5000:al=2500:alr=0.5:aal=5000:oh=3000:ohr=0.3
corr_rd2rd_options=-X -g3000 -w30 -k19 -m100 -r500 -f 0.002 -K8G -I
8G
corr_output_coverage=60
align_block_size=4000000000
align_rd2rd_options=-X -g3000 -w30 -k19 -m100 -r500 -f 0.002 -K8G -I
8G
align_filter_options=--
filter0=l=5000:aal=6000:aalr=0.5:oh=3000:ohr=0.3 --task=extend --
filter1=oh=50:ohr=0.01  --aligner diff:s=100 --min_identity 0.90
asm1_assemble_options= --min_identity 0.99 --min_coverage 1

phase_method=2
phase_rd2ctg_options=-x map-hifi  -c -p 0.5 -r 1000 -K8G
phase_use_reads=1
phase_phase_options= --coverage lc=8 --phase_options
icr=0.02:icc=3:sc=4 --
filter=i=95.00:alr=0.80:oh=100:ohr=0.01:ilid=100

phase_clair3_command= singularity exec --containall -B `pwd -P`:`pwd
-P` -B /tmp:/tmp clair3_v0.1-r12.sif /opt/bin/run_clair3.sh
phase_clair3_use_reads=0
phase_clair3_options=--platform=hifi --model_path=/opt/models/hifi  -
-include_all_ctgs
phase_clair3_rd2ctg_options=-x map-hifi  -c -p 0.5 -r 1000 -K8G
phase_clair3_phase_options=--coverage lc=8 --phase_options
icr=0.02:icc=2:sc=4 --filter i=95
phase_clair3_filter_options=

asm2_assemble_options= --reducer0 "best:cmp=2,0.1,0.1|phase:sc=2" --
min_identity 0.99 --max_trivial_length 10000 --contig_format
dual,prialt --min_coverage 1

polish_map_options=-x map-hifi -I8G -K8G -a
polish_filter_options=--filter0 oh=500:ohr=0.05:i=98
```

```
polish_cns_options=
```

In the above parameters, `$READS` is the path of the reads file. `$GENOME_ SIZE` is the genome size of the species.

Supplementary Note 3: Evaluating heterozygosity.

We used genomescope2.0[13] and jellyfish (2.3.0)[14] to evaluate the heterozygosity of these species with the following commands.

```
jellyfish count -C -m 21 -t 24 -s 4000000000 *.fastq -o reads.jf
jellyfish histo -t 24 reads.jf > reads.histo
genomescope.R -i reads.histo -o out -k 21
```

We used 60X offspring's Illumina short sequences or combined 30X paternal and maternal Illumina short sequences as input data.

Supplementary Note 4: Evaluating assemblies with merqury

We followed the instructions on the website https://github.com/marbl/merqury and used the following command to build meryl databases.

```
sh $MERQURY/best_k.sh $GENOME_SIZE
meryl k=$k count output f1.meryl f1.fastq.gz
meryl k=$k count output maternal.meryl maternal.fastq.gz
meryl k=$k count output paternal.meryl paternal.fastq.gz
sh $MERQURY/trio/hapmers.sh maternal.meryl paternal.meryl f1.meryl
```

Then, we used the following command to evaluate the assemblies.

```
$MERQURY/merqury.sh f1.meryl maternal.meryl paternal.meryl
(pri|hap1).fasta (alt|hap2).fasta $PREFIX
```

Merqury counted the number of parental-specific k-mers. We calculated the hamming error rate of the assemblies from the output of merqury using the following command.

```
$PECAT/build/bin/fxtools.py fx_hamming_error $PREFIX.hapmers.count
```

Supplementary Note 5: Evaluating gene completeness

BUSCO (5.2.2)[15] was run to evaluate the gene completeness of assemblies for all species. We used the following script:

```
busco -i $CONTIG -m geno --cpu $THREADS -l $LIBS --offline -o
contigs.busco --out_path $XXX
```

where `$CONTIG` was set to one of the assemblies and `$LIBS` was set to the corresponding OrthoDB v10 dataset. We used datasets saccharomycetes, brassicales, diptera, cetartiodactyla, and primates for *S. cerevisiae*, *A. thaliana*, *D. melanogaster*, *B. taurus,* and HG002, respectively. Those datasets can be downloaded from https://busco-data.ezlab.org/v5/data/lineages/.

Supplementary Note 6: Evaluating assemblies with Pomoxis.

The program assess_assembly in Pomoxis (https://github.com/nanoporetech/pomoxis) was used to evaluate the quality of the assemblies with the following commands:

```
cat paternal_reference.fna maternal_reference.fna > all_reference.fna
assess_assembly -r all_reference.fna -i primary.fasta
assess_assembly -r all_reference.fna -i alternate.fasta
```

Supplementary Note 7: Evaluating assembly quality with QUAST.

QUAST (5.0.2)[16] run with the following commands:

```
cat paternal_ref.fna maternal_ref.fna > all_ref.fna
cat primary.fasta alternate.fasta > all_ctgs.fasta
quast.py -r all_ref.fna all_ctgs.fasta --min-contig 5000 --large --
min-identity 90
```

For *B. taurus* (Angus×Brahman), *B. taurus* (Bison × Simmental), and HG002, the parameter min-contig was set to 50000.

Supplementary Note 8: Evaluating SNP, INDEL, and SV in HG002 assemblies

We used dipcall (v0.3)[17] and hap.py(v0.3.15)[18] to calculate the precision, recall and F1-score of the small variants (SNP and INDEL) in HG002 assemblies with following commands:

```
run-dipcall
GCA_000001405.15_GRCh38_no_alt_plus_hs38d1_analysis_set.fna
haplotype_1.fasta haplotype_2.fasta -t 48 > prefix.mak
make -j2 -f prefix.mak
```

```
hap.py HG002_GRCh38_GIAB_highconf_CG-Illfb-IllsentieonHC-Ion-10XsentieonHC-SOLIDgatkHC_CHROM1-22_v.3.3.2_highconf_triophased.vcf.gz prefix.dip.vcf.gz -f HG002_GRCh38_GIAB_highconf_CG-Illfb-IllsentieonHC-Ion-10XsentieonHC-SOLIDgatkHC_CHROM1-22_v.3.3.2_highconf_noinconsistent.bed -r GCA_000001405.15_GRCh38_no_alt_plus_hs38d1_analysis_set.fna -o output -engine=vcfeval
```

We used hapdiff (https://github.com/KolmogorovLab/hapdiff) and truvari[19] to calucate the precision, recall, and F1-score of the structural variant (SV) in HG002 assemblies with the following commands:

```
hapdiff.py --reference hs37d5.fa --pat primary.fasta --mat alternate.fasta --out-dir out -t 48
truvari bench -b HG002_SVs_Tier1_v0.6.vcf.gz -c out/hapdiff_phased.vcf.gz -o result_phased --includebed HG002_SVs_Tier1_v0.6.bed -f hs37d5.fa.gz -r 2000 --chunksize 2000 –passonly
```

The standard SV set is available from GIAB[20]. It can be downloaded from at https://ftp-trace.ncbi.nlm.nih.gov/giab/ftp/data/AshkenazimTrio/analysis/NIST_SVs_Integration_v0.6

Supplementary Figures

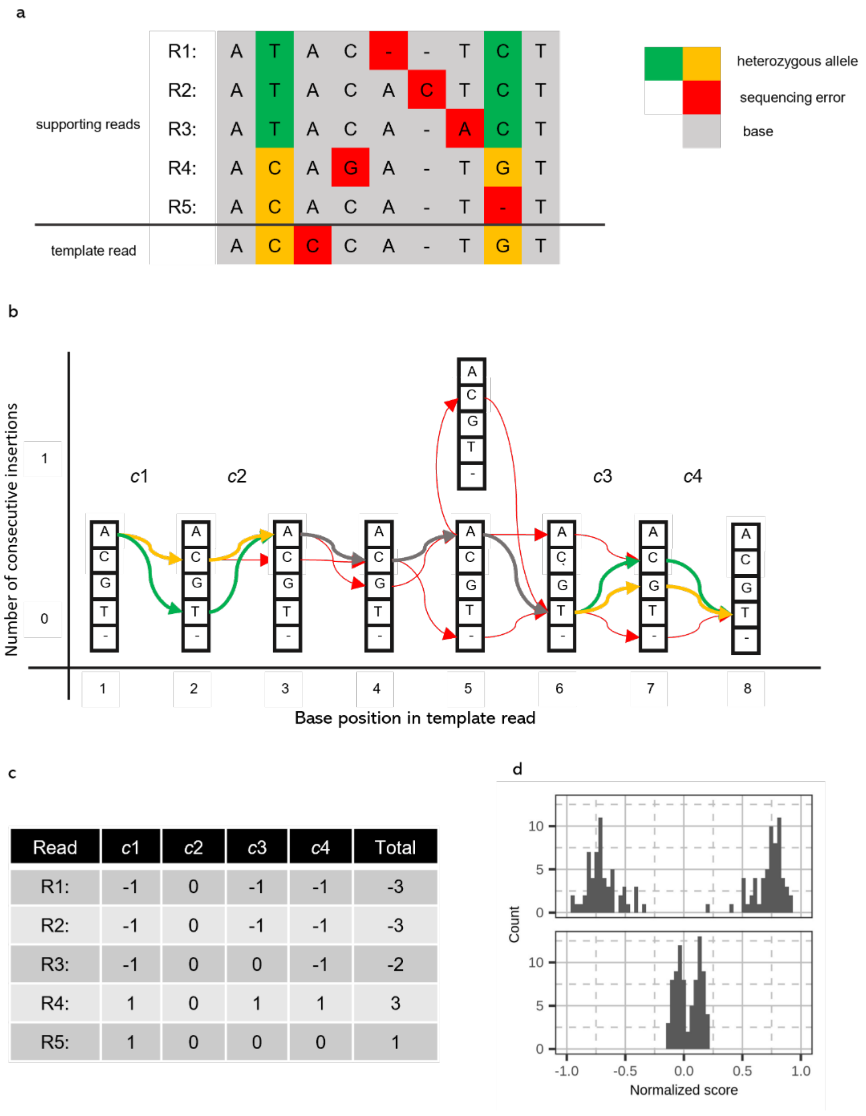

Read	*c*1	*c*2	*c*3	*c*4	Total
R1:	-1	0	-1	-1	-3
R2:	-1	0	-1	-1	-3
R3:	-1	0	0	-1	-2
R4:	1	0	1	1	3
R5:	1	0	0	0	1

Supplementary Figure 1. Illustration of haplotype-aware error correction. (**a**) The alignments between the template read and the five supporting reads R1-5. The heterozygous alleles and sequencing errors are marked with different colors. (**b**) The POA graph generated from the alignments. The red edges are supported by only one read, and they are considered sequencing errors. Other thick edges are supported by multiple reads. The edges passing through the heterozygous alleles are marked with corresponding colors. There four important locations *c*1-4 are identified in the POA graph, at which there are two dominant edges. (**c**) The scores of the supporting reads at important locations. If the supporting read and the template read pass through the same dominant edge, the score is 1 and if they pass through the different dominant edges, the score is -1. If one of them doesn't pass through any dominant edge, which means there is a sequencing error, the score is 0. (**d**) The histograms of normalized scores of two supporting reads from the HG002 dataset. Two peaks indicate that the supporting reads are from two different haplotypes. PECAT selects the reads whose scores fall into the first peak for error correction.

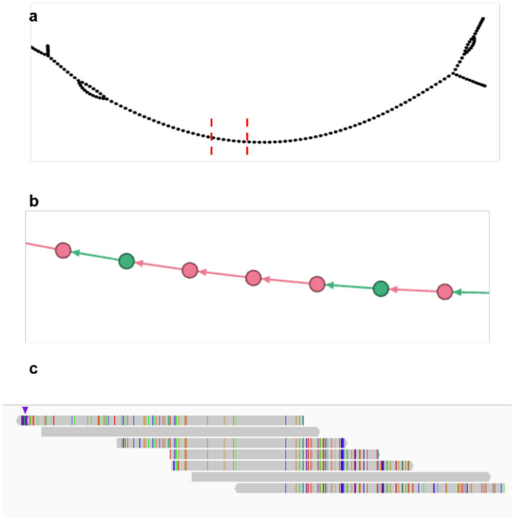

Supplementary Figure 2. Inconsistent overlaps in the string graph. (**a**) Part of the string graph built from *S. cerevisiae* (SK1 × Y12) corrected reads. (**b**) Enlarged view of the red dashed area in **a**. Each node represents a read and is colored according to its haplotype. (**c**) The overlaps between the reads in **b**. Colored dashes indicate haplotype differences.

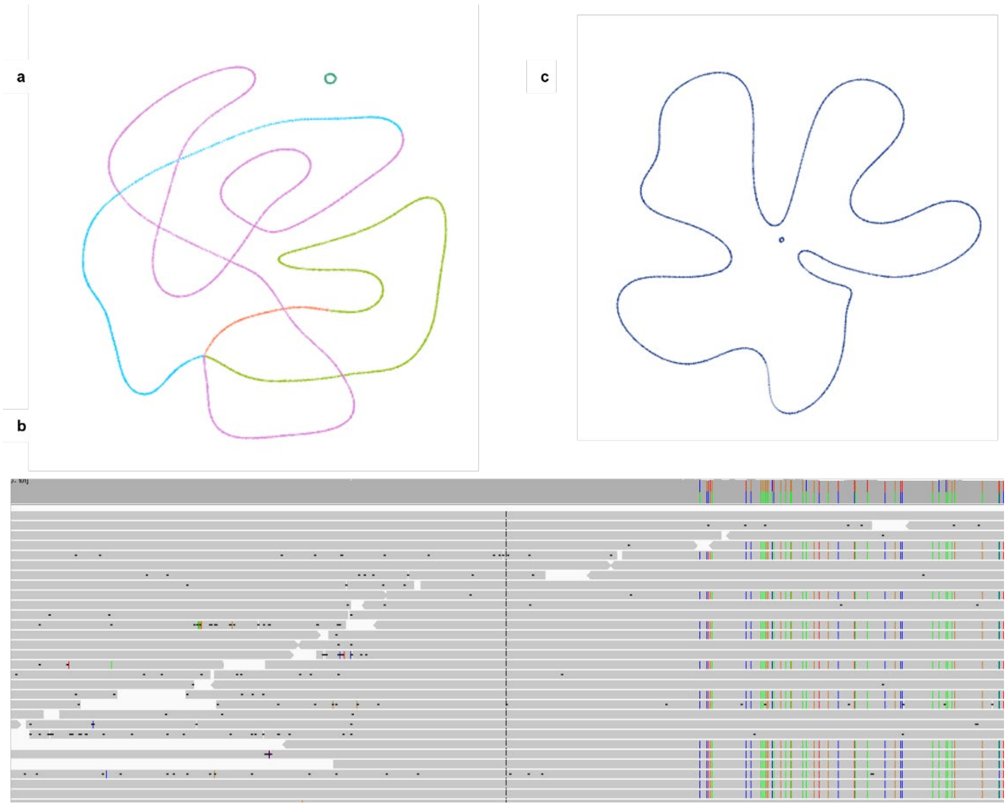

Supplementary Figure 3. Illustration of PECAT resolving the repeat of NCTC9006. (**a**) The assembly graph generated by PECAT in the first round of assembly. Different colors represent different contigs. There is an unresolvable double repeat in the graph. (**b**) The alignment between the corrected reads and the repeat. Colored dashed indicate differences. PECAT identifies the inconsistent overlaps according to the differences. (**c**) The assembly graph generated by PECAT in the second round of assembly. PECAT resolves the repeat by filtering out inconsistent overlaps in the second round of assembly.

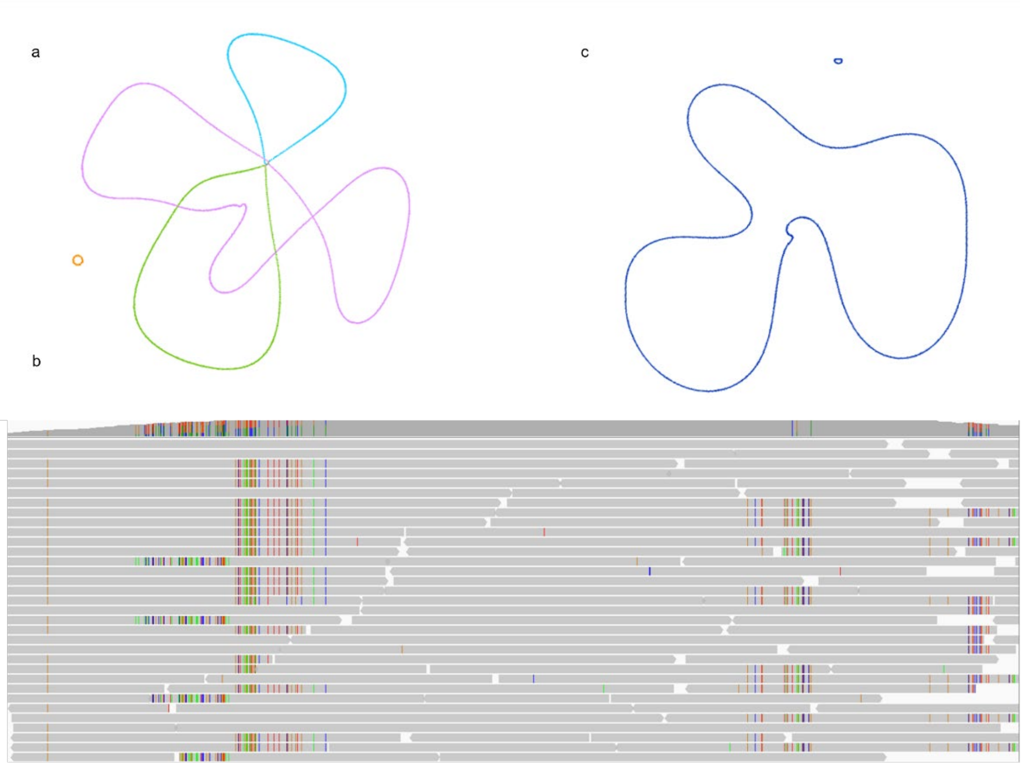

Supplementary Figure 4. Illustration of PECAT resolving the repeat of NCTC9024. (**a**) The assembly graph generated by PECAT in the first round of assembly. Different colors represent different contigs. There is an unresolvable triple repeat in the graph. (**b**) The alignments between the corrected reads and the repeat. Colored dashed indicate differences. PECAT identifies the inconsistent overlaps according to the differences. (**c**) The assembly graph generated by PECAT in the second round of assembly. PECAT resolves the repeat by filtering out inconsistent overlaps in the second round of assembly.

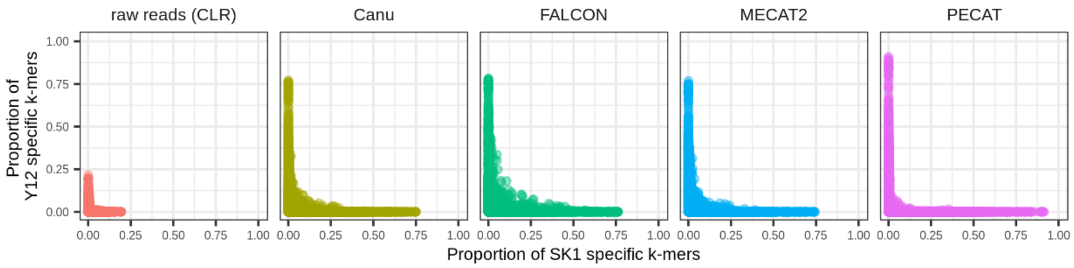

Supplementary Figure 5. Haplotype-specific k-mers consistency of *S. cerevisiae* (SK1 × Y12) raw reads and corrected reads by different methods. Each point corresponds to a read. Its coordinate gives the proportion of the parental specific k-mers in the read, where k is 17. All 40X longest reads are shown in each subfigure.

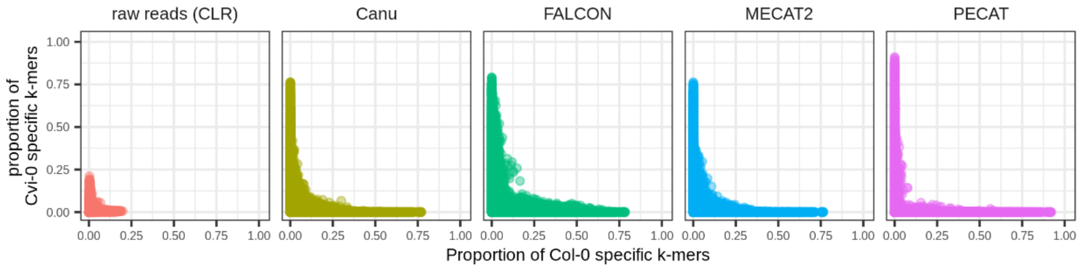

Supplementary Figure 6. Haplotype-specific k-mers consistency of *A. thaliana* (Col-0 × Cvi-0) raw reads and corrected reads by different methods. Each point corresponds to a read. Its coordinate gives the proportion of the parental specific k-mers in the read, where k is 18. All 40X longest reads are shown in each subfigure.

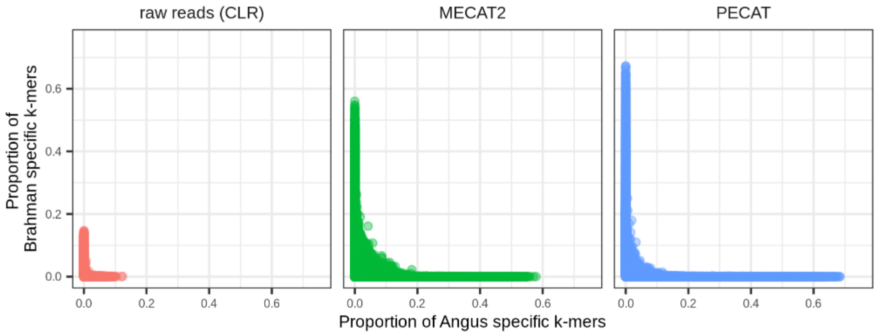

Supplementary Figure 7. Haplotype-specific k-mers consistency of *B. taurus* (Angus×Brahman) raw reads and corrected reads by different methods. Each point corresponds to a read. Its coordinate gives the proportion of the parental specific k-mers in the read, where k is 21. All 40X longest reads are shown in each subfigure.

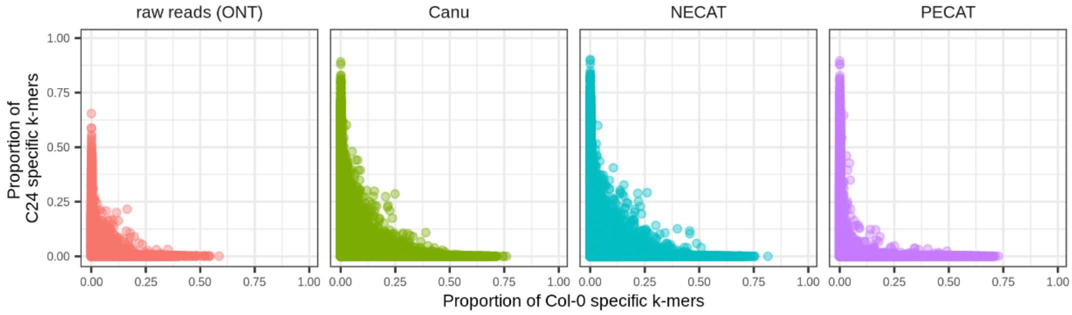

Supplementary Figure 8. Haplotype-specific k-mers consistency of *A. thaliana* (Col-0×C24) raw reads and corrected reads by different methods. Each point corresponds to a read. Its coordinate gives the proportion of the parental specific k-mers in the read, where k is 18. All 40X longest reads are shown in each subfigure.

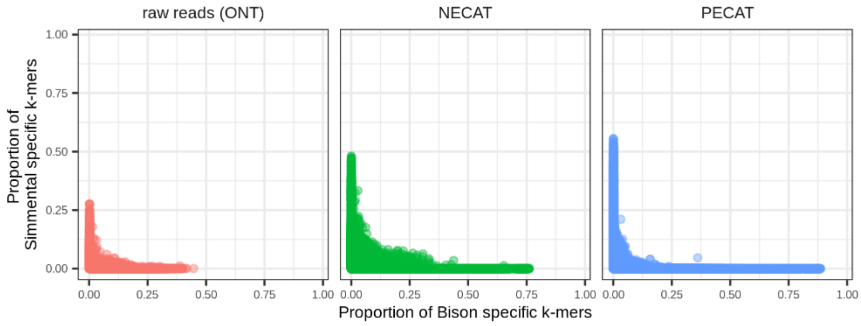

Supplementary Figure 9. Haplotype-specific k-mers consistency of *B. taurus* (Bison × Simmetal) raw reads and corrected reads by different methods. Each point corresponds to a read. Its coordinate gives the proportion of the parental specific k-mers in the read, where k is 21. All 40X longest reads are shown in each subfigure.

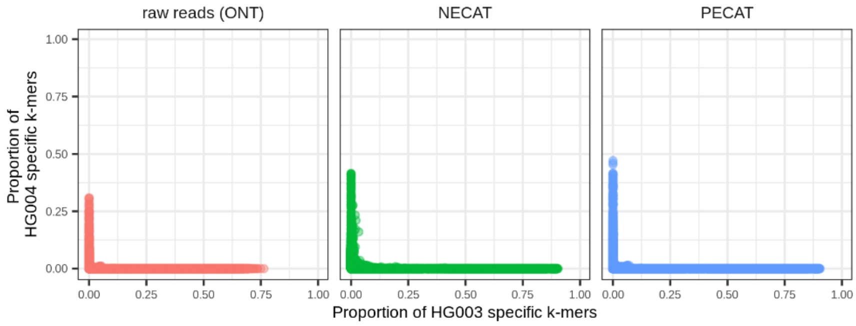

Supplementary Figure 10. Haplotype-specific k-mers consistency of HG002 R9 raw reads and corrected reads by different methods. (**a**) Each point corresponds to a read. Its coordinate gives the proportion of the parental specific k-mers in the read, where k is 21. All 40X longest reads are shown in each subfigure.

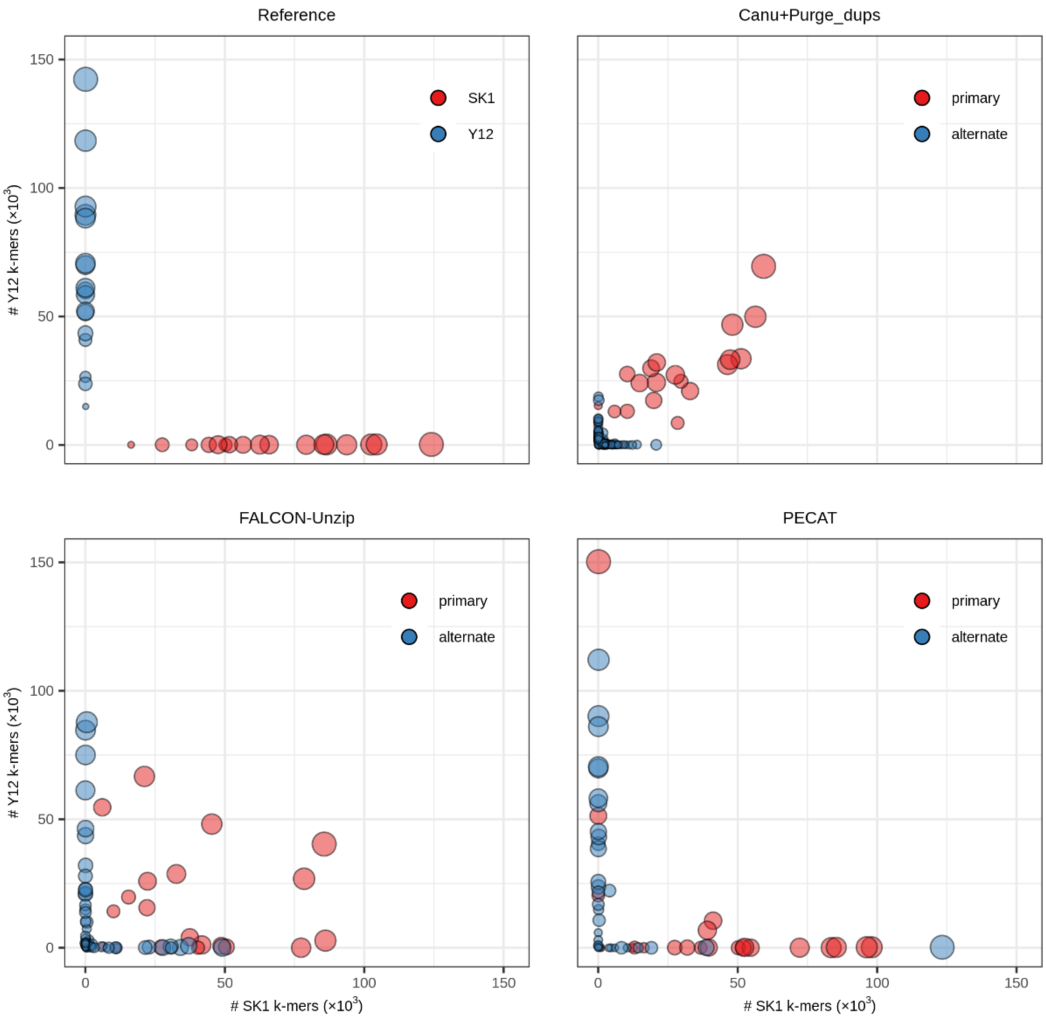

Supplementary Figure 11. Haplotype-specific k-mer blob plots of the *S. cerevisiae* (SK1 × Y12) reference genome and assemblies by different methods. All assemblies are in the primary/alternate format. Each blob corresponds to a contig. The coordinate of the blob gives the count of the parental specific k-mers in the contig, where k is 17. Blob size is proportional to contig length.

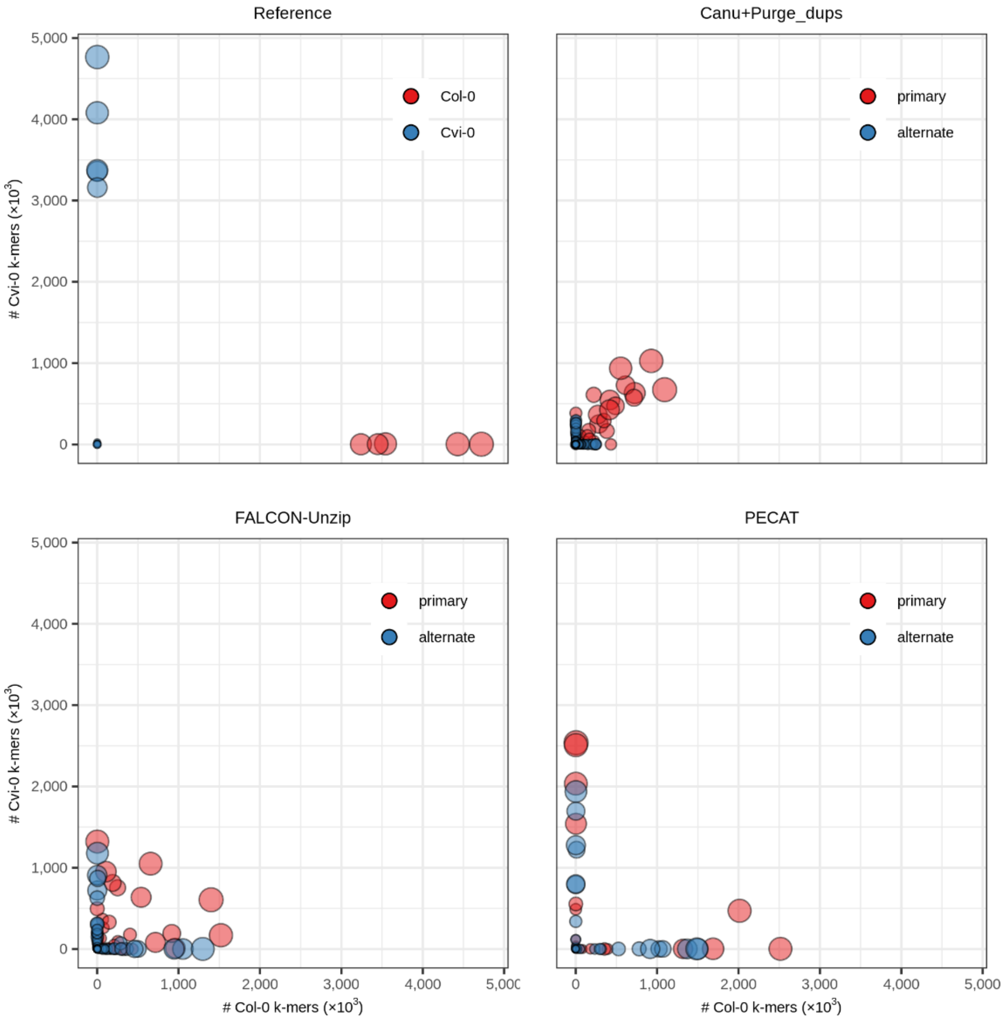

Supplementary Figure 12. Haplotype-specific k-mer blob plots of the *A. thaliana* (Col-0 × Cvi-0) reference genome and assemblies by different methods. All assemblies are in the primary/alternate format. Each blob corresponds to a contig. The coordinate of the blob gives the count of the parental specific k-mers in the contig, where k is 18. Blob size is proportional to contig length.

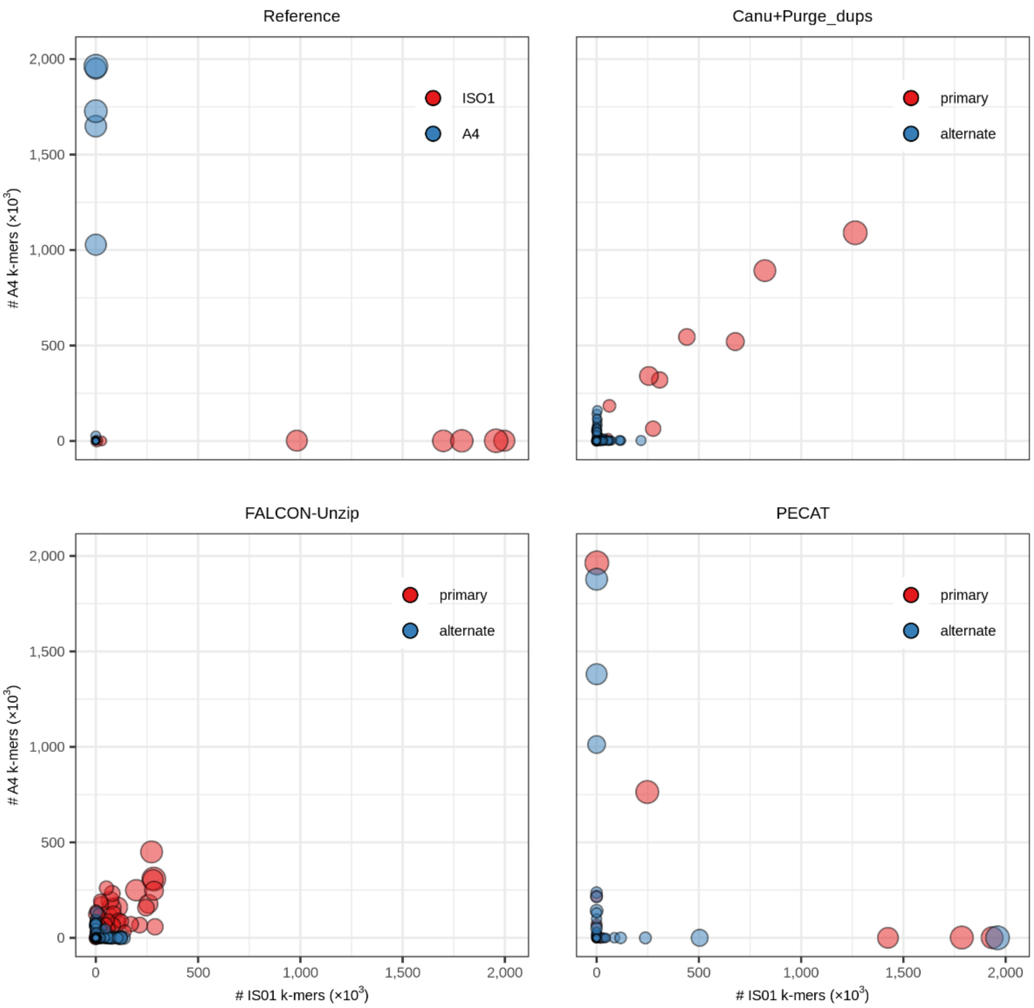

Supplementary Figure 13. Haplotype-specific k-mer blob plots of the *D. melanogaster* (ISO1 × A4) reference genome and assemblies by different methods. All assemblies are in the primary/alternate format. Each blob corresponds to a contig. The coordinate of the blob gives the count of the parental specific k-mers in the contig, where k is 18. Blob size is proportional to contig length.

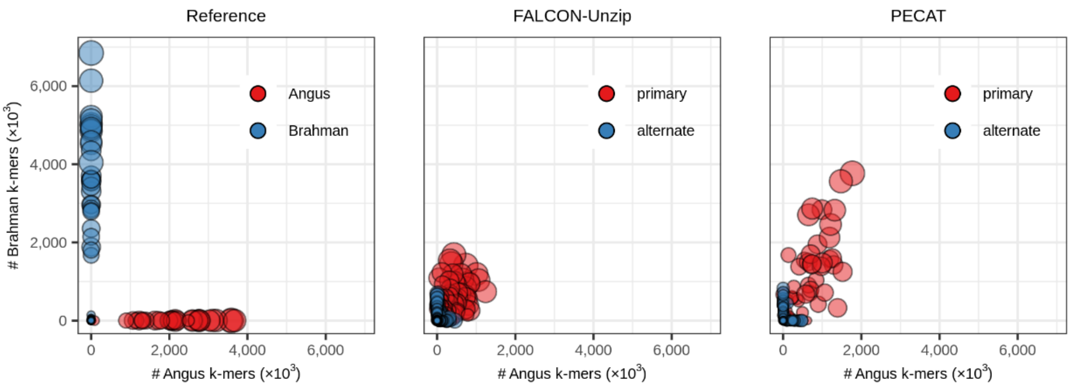

Supplementary Figure 14. Haplotype-specific k-mer blob plots of the *B. taurus* (Angus×Brahman) reference genome and assemblies by different methods. All assemblies are in the primary/alternate format. Each blob corresponds to a contig. The coordinate of the blob gives the count of the parental specific k-mers in the contig, where k is 21. Blob size is proportional to contig length.

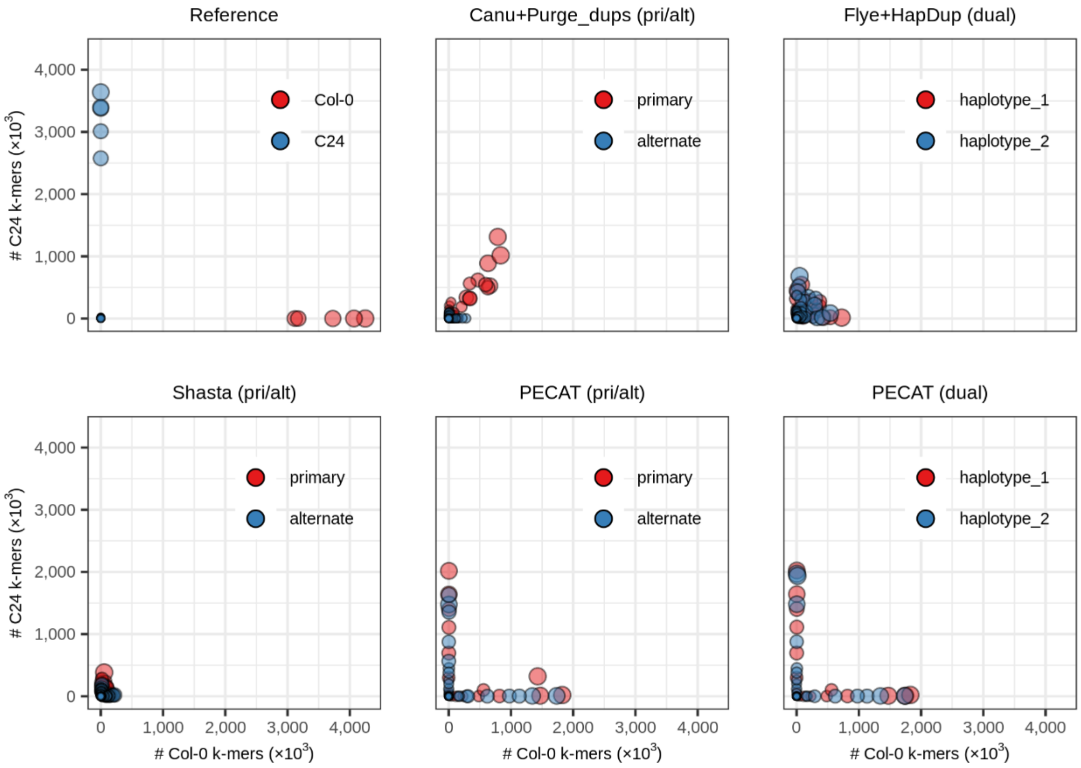

Supplementary Figure 15. Haplotype-specific k-mer blob plots of the *A. thaliana* (Col-0 × C24) reference genome and assemblies by different methods. pri/alt or dual represents that the assembly is in the primary/alternate format or the dual assembly format. Each blob corresponds to a contig. The coordinate of the blob gives the count of the parental specific k-mers in the contig, where k is 18. Blob size is proportional to contig length.

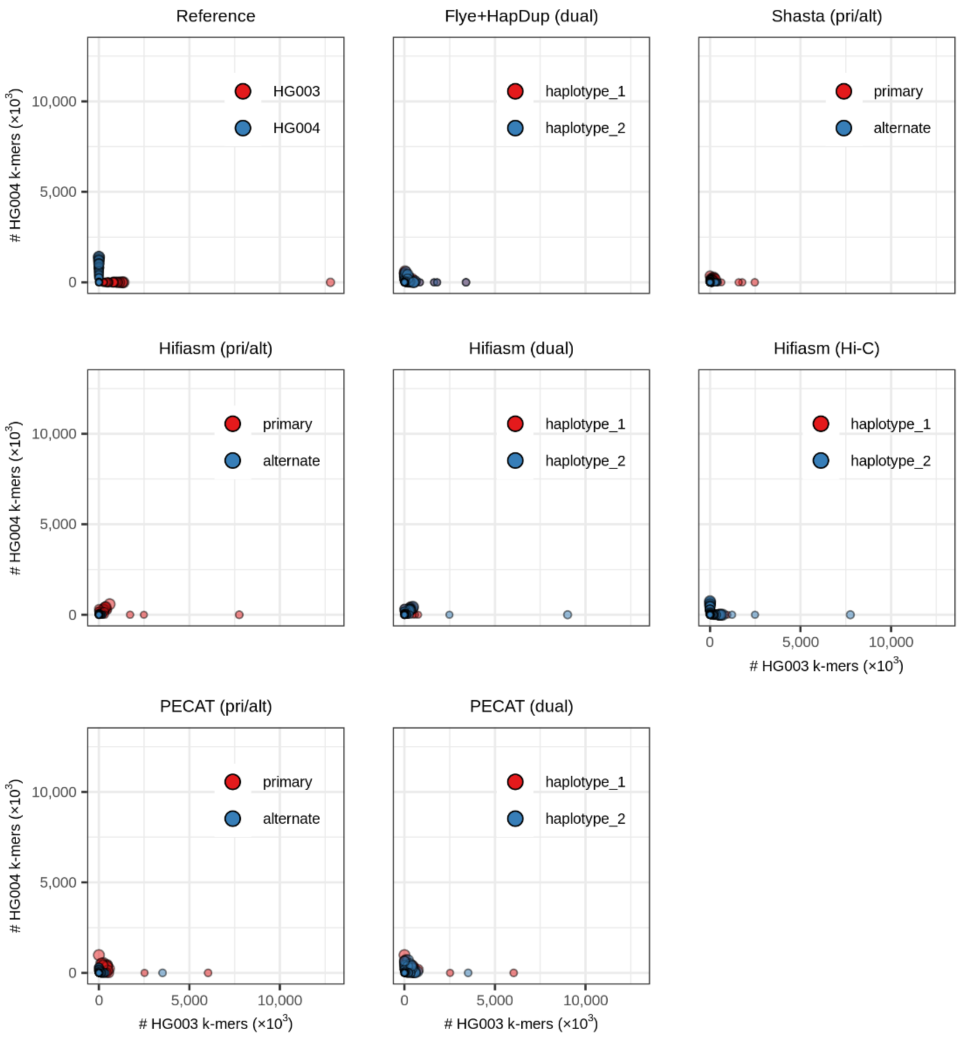

Supplementary Figure 16. Haplotype-specific k-mer blob plots of the HG002 reference genome and assemblies by different methods. The assemblies By Hifiasm are from HiFi reads and the others are from Nanopore R9 (ultra-long) reads. Hi-C represents that the assembly uses the additional Hi-C reads. pri/alt or dual represents that the assembly is in the primary/alternate format or the dual assembly format. Each blob corresponds to a contig. The coordinate of the blob gives the count of the parental specific k-mers in the contig, where k is 21. Blob size is proportional to contig length.

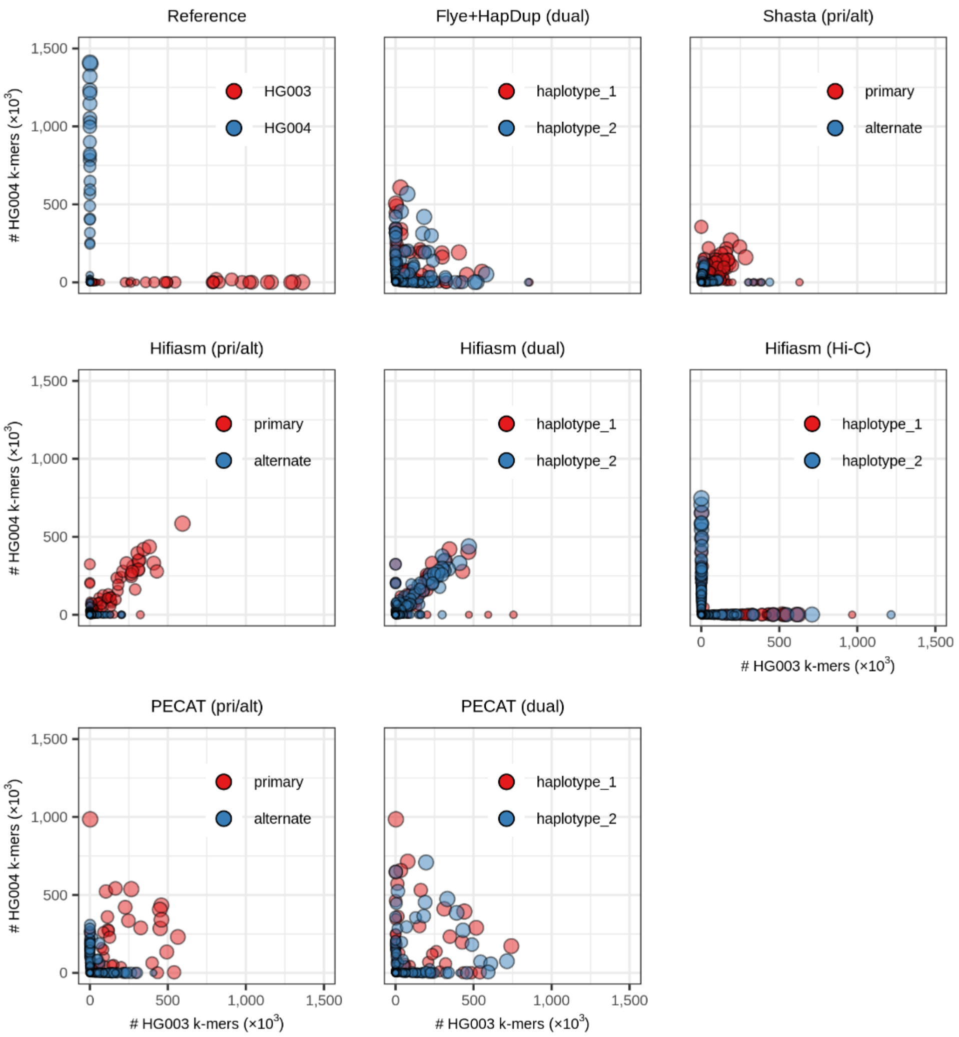

Supplementary Figure 17. Enlarged view of **Supplementary Figure 16**.

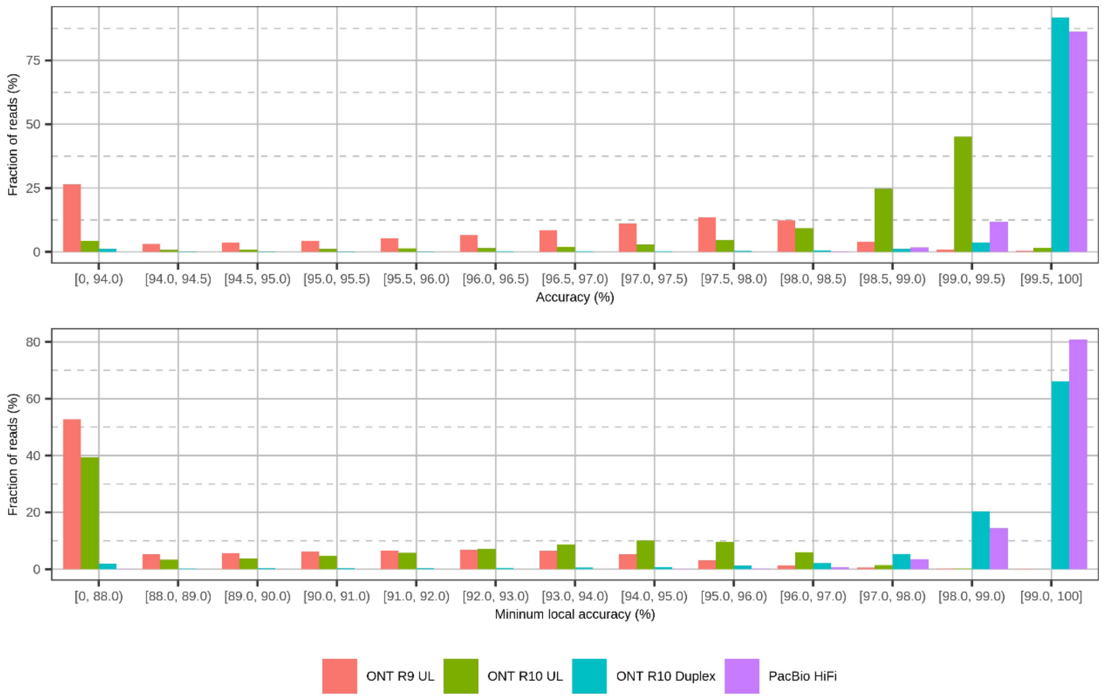

Supplementary Figure 18. Histogram plot for accuracy of HG002 reads using different sequencing techniques. ‘Minimum local accuracy’ is the minimum accuracy of windows with 1000 bp in a read. ‘ONT’ indicates the dataset is composed of Nanopore reads. ‘UL’ indicates the reads are ultra-long reads. ‘Duplex’ indicates the dataset is generated by the duplex sequencing method.

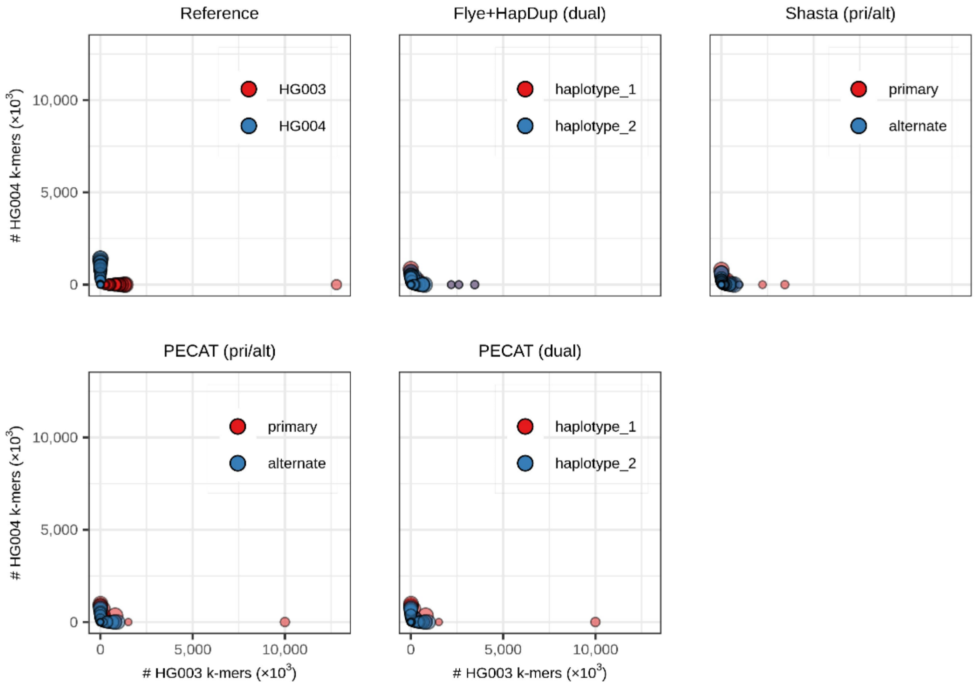

Supplementary Figure 19. Haplotype-specific k-mer blob plots of the HG002 reference genome and assemblies by different methods from Nanopore R10 (ultra-long) reads. pri/alt or dual represents that the assembly is in the primary/alternate format or the dual assembly format. Each blob corresponds to a contig. The coordinate of the blob gives the count of the parental specific k-mers in the contig, where k is 21. Blob size is proportional to contig length.

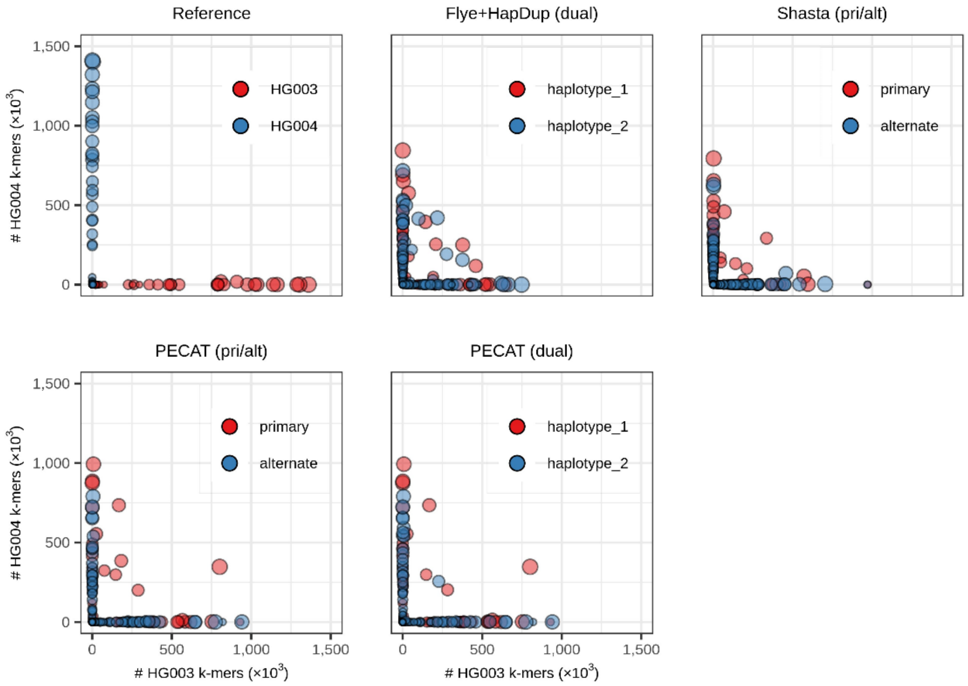

Supplementary Figure 20. Enlarged view of **Supplementary Figure 19**.

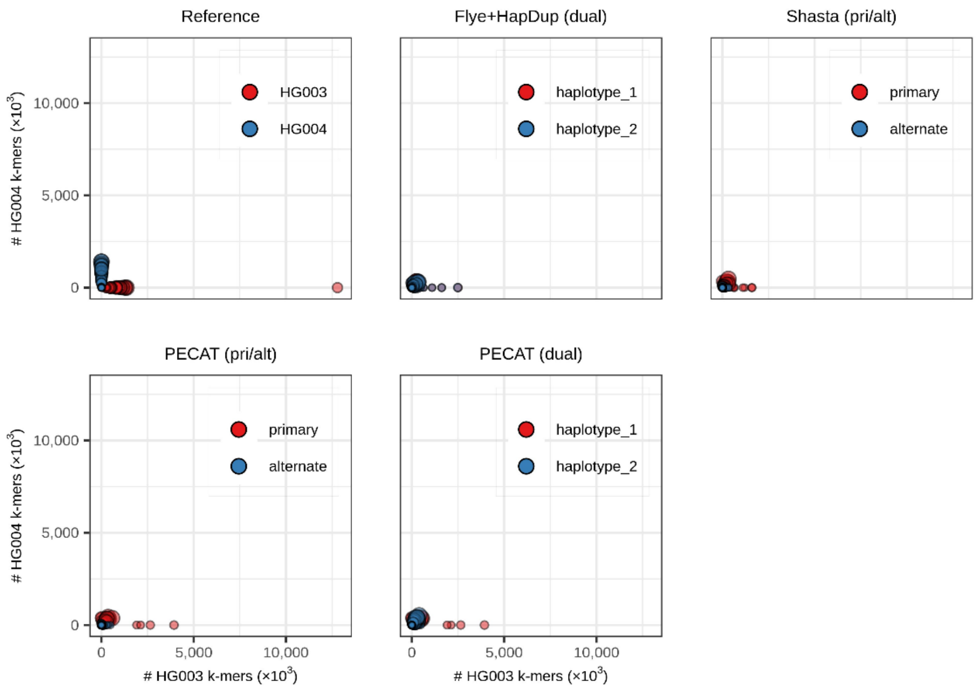

Supplementary Figure 21. Haplotype-specific k-mer blob plots of the HG002 reference genome and assemblies by different methods from Nanopore R10 duplex reads. pri/alt or dual represents that the assembly is in the primary/alternate format or the dual assembly format. Each blob corresponds to a contig. The coordinate of the blob gives the count of the parental specific k-mers in the contig, where k is 21. Blob size is proportional to contig length.

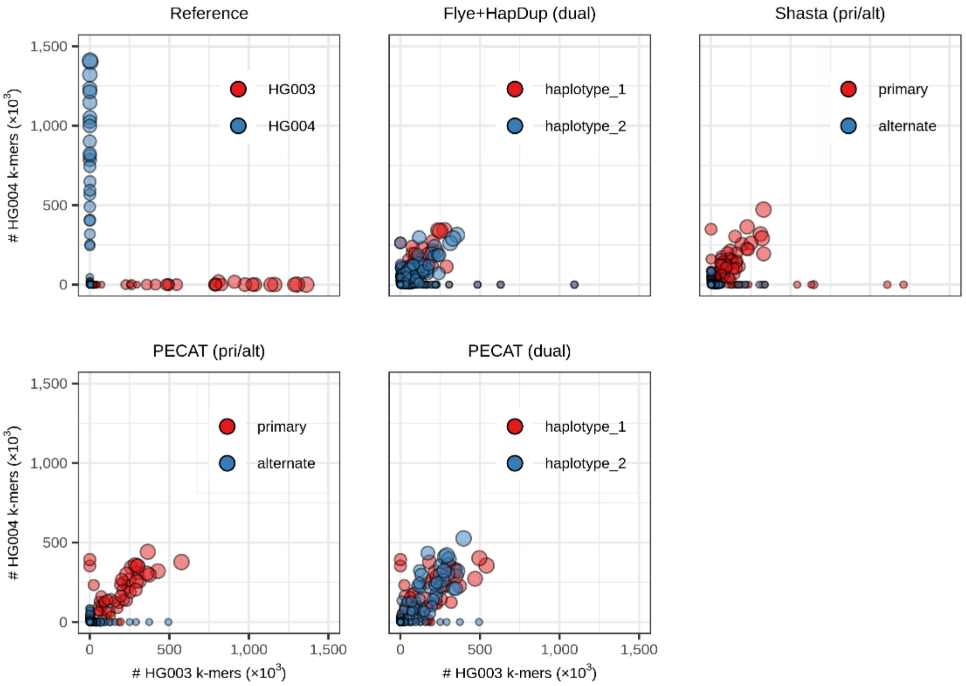

Supplementary Figure 22. Enlarged view of **Supplementary Figure 22**.

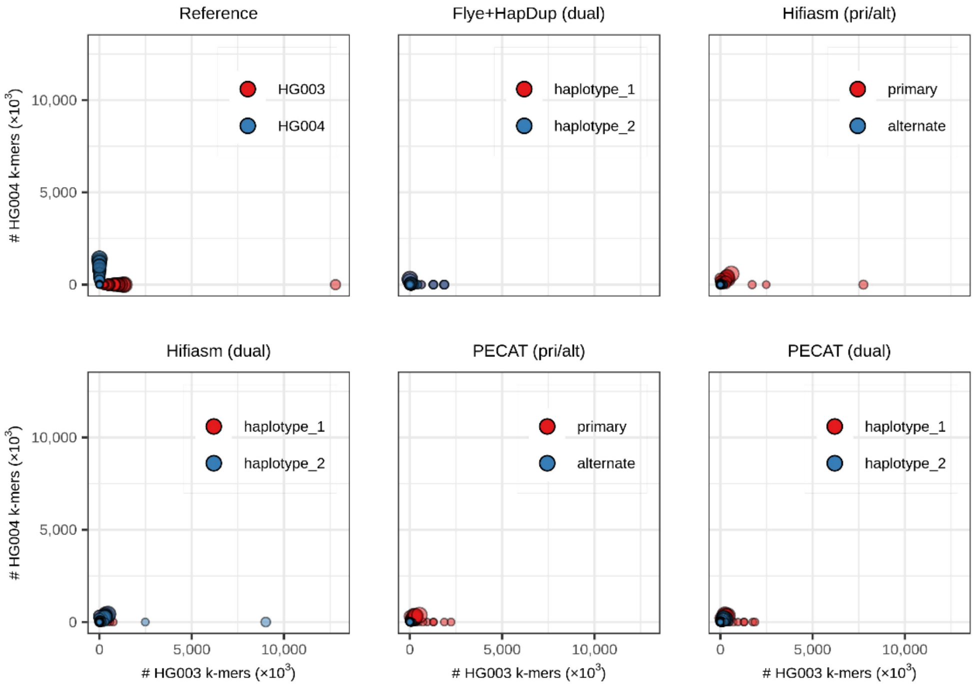

Supplementary Figure 23. Haplotype-specific k-mer blob plots of the HG002 reference genome and assemblies by different methods from PacBio HiFi reads. pri/alt or dual represents that the assembly is in the primary/alternate format or the dual assembly format. Each blob corresponds to a contig. The coordinate of the blob gives the count of the parental specific k-mers in the contig, where k is 21. Blob size is proportional to contig length.

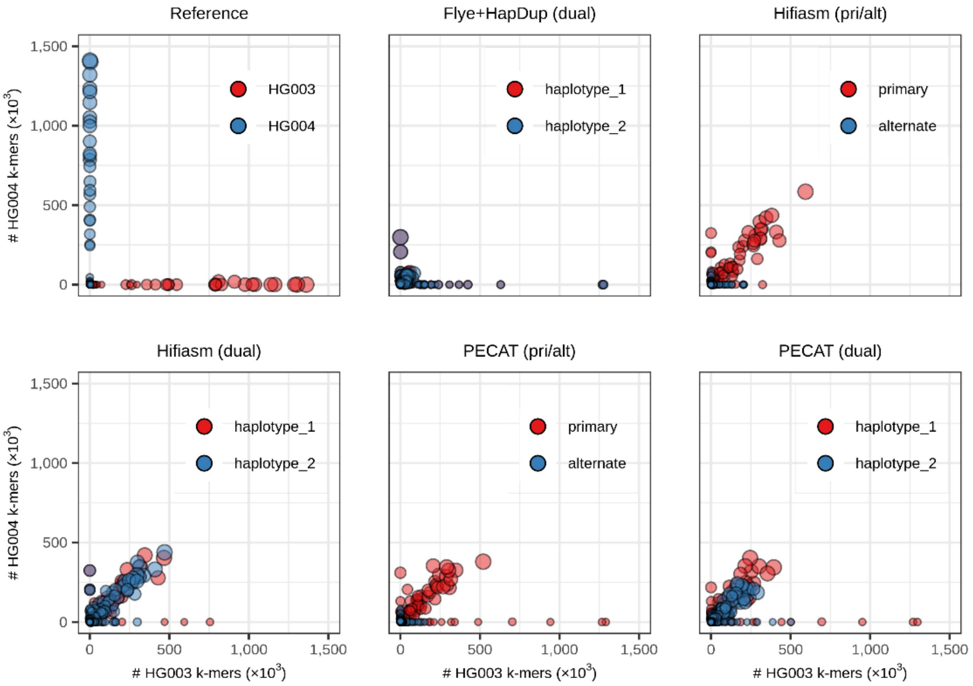

Supplementary Figure 24. Enlarged view of **Supplementary Figure 24**.

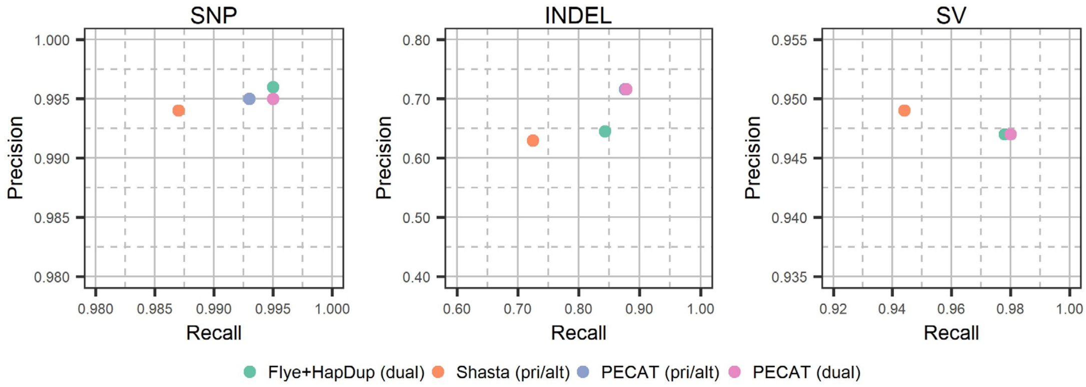

Supplementary Figure 25. Precisions and recalls of small variants (SNP, INDEL) and structural variants (SV) in HG002 assemblies from Nanopore R10 (ultra-long) reads. 'pri/alt' represents primary/alternate format. 'dual' represents dual assembly format. The points of PECAT (pri/alt) and PECAT (dual) overlap in the right two subfigures.

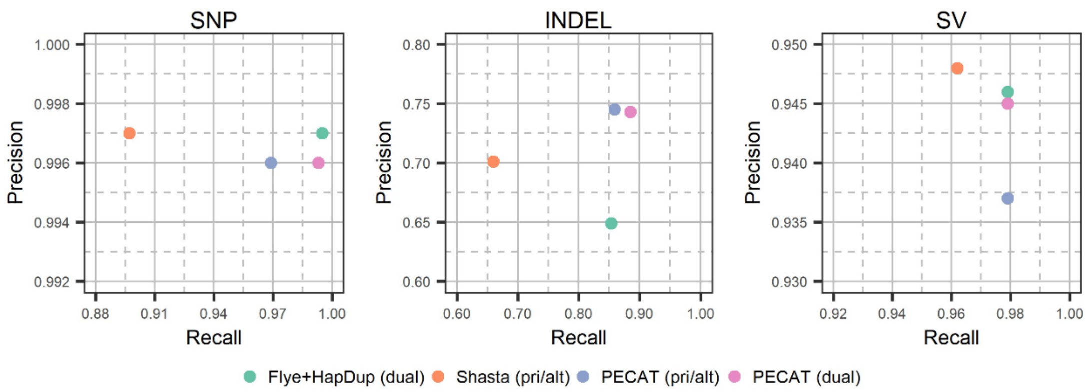

Supplementary Figure 26. **Precisions and recalls of small variants (SNP, INDEL) and structural variants (SV) in HG002 assemblies from Nanopore R10 duplex reads.** 'pri/alt' represents primary/alternate format. 'dual' represents dual assembly format.

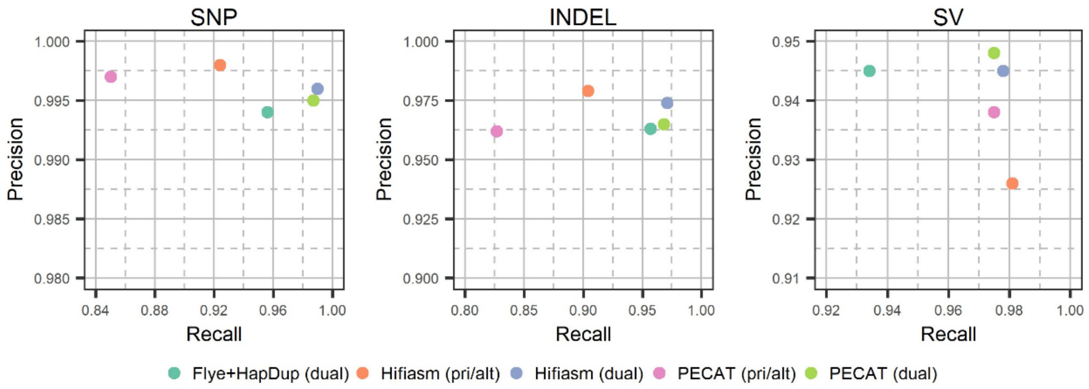

Supplementary Figure 27. **Precisions and recalls of small variants (SNP, INDEL) and structural variants (SV) in HG002 assemblies from PacBio HiFi reads.** ‘pri/alt’ represents primary/alternate format. ‘dual’ represents dual assembly format.

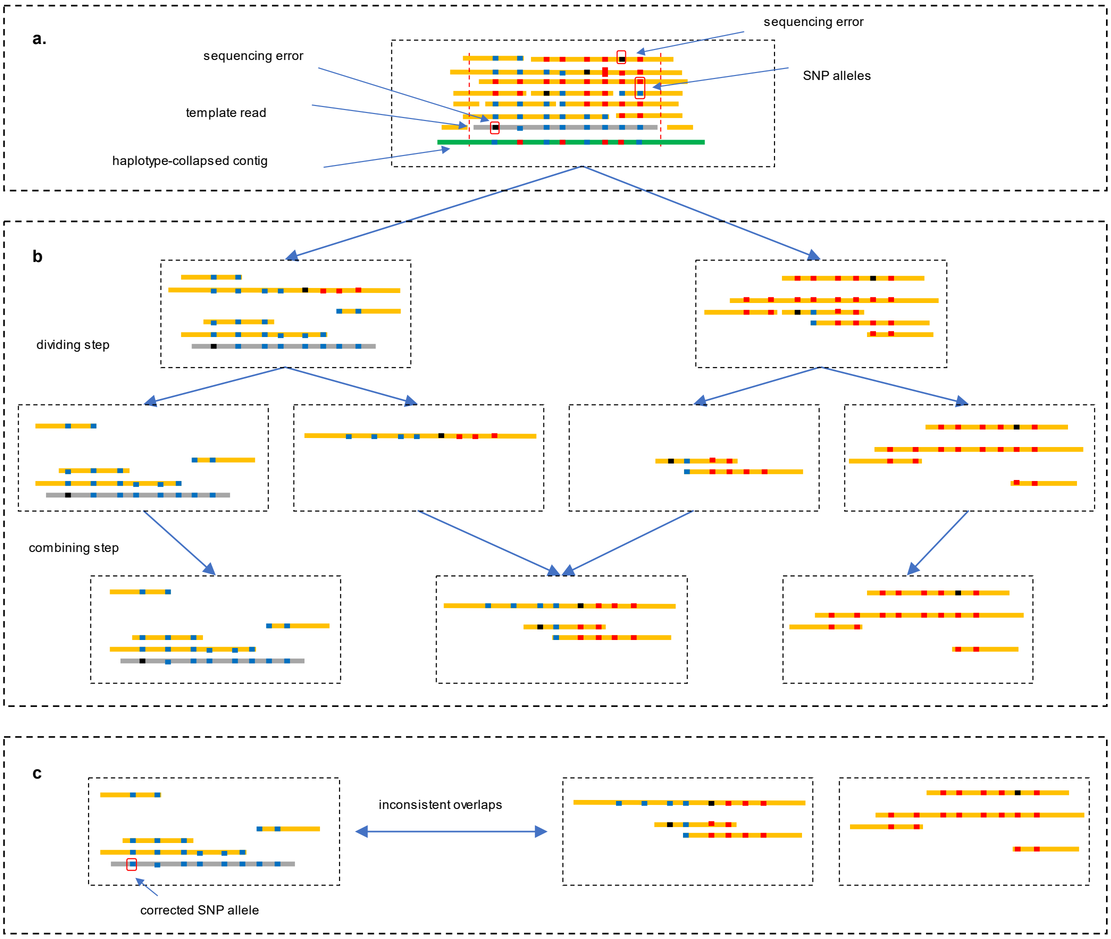

Supplementary Figure 28. Illustration of identifying inconsistent overlaps. (**a**) The query reads are collected for each template read. (**b**) A divide-then-combine strategy is used to cluster reads. (**c**) SNP alleles in the template reads are corrected. The inconsistent overlaps between the template read and the query reads are identified.

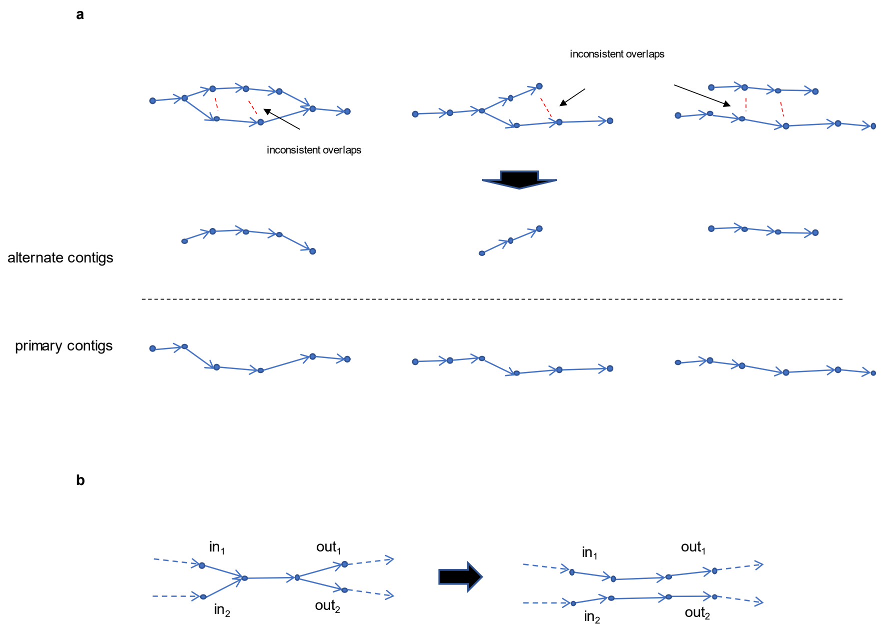

Supplementary Figure 29. Illustration of generating two sets of contigs. (a) Three structures are found in the string graph. The two paths are identified in each structure, corresponding to the primary contig and the alternate contig respectively. The red dashed lines mean that there are inconsistent overlaps between the reads in the two paths. **(b)** Phasing two adjacent bubble structures for dual assembly format.

Supplementary Tables

Supplementary Table 1. Performance of supporting read selection method of PECAT

Sample	*S. cerevisiae* SK1 × Y12 (CLR, 200X)	*A. thaliana* Col-0 × Cvi-0 (CLR, 164X)	*D. melanogaster* ISO1 × A4 (CLR, 200X)	*B. taurus* Angus × Brahman (CLR, 135X)	*A. thaliana* Col-0 × C24 (ONT, 106X)	*B. taurus* Bison × Simmental (ONT, 200X)	HG002 (ONT, R9, 59X)
Heterozygosity rate (%)	0.85	1.04	0.84	1.12	0.83	1.48	0.34
Percentage of selected reads in all supporting reads (%)	56.8	63.9	54.8	54.5	56.7	53.6	56.5
Percentage of inconsistent reads in all supporting reads (%)	36.7	31.5	41.1	28.1	33.8	37.2	27.4
Percentage of inconsistent reads in selected reads (%)	2.8	3.5	4.9	3.6	4.3	2.9	4.2
Percentage of inconsistent reads in selected reads after re-weighting the scores (%)	2.1	3.1	4.0	3.1	3.5	2.3	3.4

'CLR' indicates there are PacBio CLR reads. 'ONT' indicates there are Nanopore reads. 'R9' indicates it is Nanopore R9 data.

Supplementary Table 2. Performance comparison of error correction on simulated data

Sample	Pipeline	Size (Mb)	Max (Kb)	N50 (Kb)	Accuracy of base (%)	Accuracy of SNP (%)
PacBio CLR het = 0.01	raw data	89.2	62.1	26.3	88.58	96.55
	Canu	81.5	56.4	25.6	99.44	80.53
	MECAT2	86.3	60.1	25.8	99.28	66.84
	PECAT	87.3	61.0	25.7	99.77	99.83
PacBio CLR het = 0.005	raw data	89.2	62.9	26.5	88.52	96.48
	Canu	81.2	56.6	25.7	99.54	76.70
	MECAT2	86.1	62.1	26.0	99.45	59.36
	PECAT	87.3	61.9	25.9	99.77	99.84
PacBio CLR het = 0.001	raw data	89.2	58.3	26.4	88.66	96.44
	Canu	82.2	54.5	25.8	99.66	73.29
	MECAT2	86.5	57.5	25.9	99.63	54.27
	PECAT	87.3	57.3	25.8	99.76	99.81
PacBio CLR het = 0.0005	raw data	89.2	59.0	26.3	88.64	96.57
	Canu	82.6	55.2	25.6	99.66	72.83
	MECAT2	86.6	58.1	25.8	99.66	54.05
	PECAT	87.3	57.9	25.7	99.75	99.46
PacBio CLR het = 0.0001	raw data	89.2	57.8	26.6	88.61	96.82
	Canu	82.2	52.2	25.9	99.67	72.31
	NECAT	86.4	56.7	26.1	99.67	54.64
	PECAT	87.3	56.5	26.0	99.78	91.83
Nanopore het = 0.01	raw data	89.2	57.6	26.1	88.59	98.14
	Canu	79.2	53.6	25.4	99.28	76.41
	NECAT	87.1	56.8	25.6	99.40	58.65
	PECAT	87.3	56.7	25.5	99.44	99.94
Nanopore het = 0.005	raw data	89.2	55.2	26.1	89.10	98.09
	Canu	80.1	50.7	25.6	99.69	73.75
	NECAT	87.2	54.2	25.7	99.77	56.67
	PECAT	87.3	54.2	25.6	99.94	99.95
Nanopore het = 0.001	raw data	89.2	59.9	26.5	89.06	98.22
	Canu	79.9	53.1	25.9	99.79	72.73
	NECAT	87.2	57.9	26.0	99.87	56.86
	PECAT	87.3	57.8	25.9	99.93	99.90
Nanopore het = 0.0005	raw data	89.2	58.1	26.6	89.10	98.07
	Canu	80.2	52.0	26.0	99.69	71.64
	NECAT	87.3	57.2	26.1	99.77	55.96
	PECAT	87.3	57.0	26.0	99.94	99.72
Nanopore het = 0.0001	raw data	89.2	62.1	26.3	89.05	98.25
	Canu	79.5	52.6	25.7	99.82	73.09

	NECAT	87.4	61.3	25.8	99.87	54.81
	PECAT	87.3	61.3	25.7	99.95	94.10

The simulated data is illustrated in **Supplementary Note 1**. ‘Size’ is the total number of base pairs of the reads. ‘Max’ is the max length of the reads. ‘N50’ is the length of the shortest read for which longer and equal-length reads cover at least 50% of the corrected reads. ‘Accuracy of base’ is the percentage of matched base pairs in the alignments between the reads and the reference genome. ‘Accuracy of SNP’ is the accuracy of SNP alleles in reads. ‘het’ represents the heterozygosity rate of the simulated data.

Supplementary Table 3. Intermediate state of the first round of assembly

Sample	Symbol / Formula	*S. cerevisiae* SK1 × Y12	*A. thaliana* Col-0 × Cvi-0	*D. melanogaster* ISO1 × A4	*B. taurus* Angus × Brahman	*A. thaliana* Col-0 × C24	*B. taurus* Bison × Simmental	HG002
Number of all candidate overlaps	N1	15765862	217154507	126571948	451388849	761657318	470660333	529956421
Number of overlaps without long overhang	N2	14931099	194873544	22549502	393535386	609033764	190990689	231772901
Number of the overlaps for constructing the string graph after removing the overlaps whose reads are contained in other reads or with low coverage	N3	21689	392687	193075	3376177	197563	2350982	344684
Number of all edges in the string graph*	2*N3	43378	785374	386150	6752354	395126	4701964	689368
Number of the transitive edges in the string graph	N4	32486	654336	323358	5502574	313326	3978100	526502
Number of low-quality edges in the string graph	N5	234	15980	5942	28094	10814	13340	11182
Number of transitive edges which are reactivated in the string graph	N6	218	802	476	5094	412	2164	850
Number of the overlaps introduced by contained reads	N7	64	1527	337	3830	1525	3063	1548
Percentage of the overlaps with long overhang in all candidate overlaps	1-N2/N1	5.3%	10.3%	82.2%	12.8%	20.0%	59.4%	56.3%
Percentage of the overlaps for constructing the string graph in the overlaps without long overhang	N3/N2	0.15%	0.18%	0.86%	0.86%	0.03%	1.23%	0.15%
Percentage of active edges in all edges in the string graph	1-N4/(2*N3)	25.1%	16.7%	16.3%	18.5%	20.7%	15.4%	23.6%
Percentage of low-quality edges in active edges in the string graph	N5/(2*N3-N4)	2.1%	12.2%	9.5%	2.2%	13.2%	1.8%	6.9%
Percentage of transitive edges that are reactivated in the string graph	N6/N4	0.67%	0.12%	0.15%	0.09%	0.13%	0.05%	0.16%

Percentage of edges introduced by contained reads	N7/N3	0.30%	0.39%	0.17%	0.11%	0.77%	0.13%	0.45%

*An overlap corresponds to two edges in the string graph. 'Symbol / Formula' is a symbol for the corresponding metric or a formula to calculate the corresponding metric.

Supplementary Table 4. Performance of inconsistent overlaps identification method of PECAT

Sample	Reads used	ASM1 (%)	ASM2 (%)
S. cerevisiae, SK1 × Y12, CLR	Corrected	36.98	0.32
A. thaliana, Col-0 × Cvi-0, CLR	Corrected	16.00	0.57
D. melanogaster, ISO1 × A4, CLR	Corrected	15.94	0.90
B. taurus, Angus × Brahman, CLR	Corrected	36.55	0.63
A. thaliana, Col-0 × C24, ONT	Corrected	16.62	1.57
	Raw	16.62	1.57
	Corrected +Raw	16.62	0.79
B. taurus, Bison × Simmental, ONT	Corrected	18.08	0.06
	Raw	18.08	0.07
	Corrected +Raw	18.08	0.03
HG002, ONT, R9	Corrected	37.45	1.46
	Raw	37.45	1.12
	Corrected+Raw	37.45	1.09

'ASM1' and 'ASM2' are the percentages of inconsistent overlaps in simplified assembly graphs in the first and the second round of assembly. 'CLR' indicates there are PacBio CLR reads. 'ONT' indicates there are Nanopore reads. 'R9' indicates it is Nanopore R9 data. 'Reads used' means which reads are used to identify inconsistent overlaps. 'Corrected' and 'Raw' represent corrected reads and raw reads, respectively.

Supplementary Table 5. Performance of assemblies before and after removing inconsistent overlaps of PECAT.

	The first round of assembly				The second round of assembly			
Sample	Size (Mb)	Count	Max (Mb)	NG50 (Mb)	Size (Mb)	Count	Max (Mb)	NG50 (Mb)
S. cerevisiae	12.9	83	1.48	0.89	12.3	24	1.5	0.8
SK1×Y12, CLR	0.9	27	0.06	0.00	11.8	33	1.5	0.8
A. thaliana	139.8	489	16.84	14.07	130.6	373	16.6	14.3
Col-0×Cvi-0, CLR	74.0	845	0.58	0.05	120.4	163	12.8	7.8
D. melanogaster	164.2	391	28.1	23.4	149.6	243	27.1	24.5
ISO1×A4, CLR	84.5	408	1.8	0.1	135.7	201	27.0	11.9
B. taurus	2762.6	3397	110.8	55.2	2744.7	2445	157.2	72.4
Angus×Brahman, CLR	128.8	1940	0.5	0.0	2447.6	3424	17.6	2.8
A. thaliana	138.6	308	15.3	13.6	131.1	136	16.8	14.3
Col-0×C24, ONT	63.7	439	1.7	0.0	123.8	95	14.8	7.7
B. taurus	2984.2	1092	156.3	72.9	2963.2	498	161.1	94.1
Bison×Simmental, ONT	1519.8	5587	1.8	0.2	2765.5	1113	158.9	93.6
HG002	3125.3	735	169.9	85.6	3058.7	200	153.3	92.9
ONT, R9	137.5	267	2.6	0.0	2857.2	418	49.4	15.0

'Size' is the total number of base pairs in all contigs generated by PECAT. 'Max' is the max length of the contigs. 'NG50' is the length of the shortest contig for which longer and equal length contigs cover at least 50% of genome size. The genome sizes of *S. cerevisiae*, *A. thaliana*, *D. melanogaster*, *B. taurus,* and HG002 that we used for evaluation are 12M, 130M, 140M, 2.7G, and 3G, respectively. The primary and alternate contigs are separately reported on the top and the bottom of each row.

Supplementary Table 6. Performance comparison of error correction

Sample	Pipeline	N50	Match (%)	Mismatch (%)	Insertion (%)	Deletion (%)	Consistency (%)	Completeness PAT (%) / MAT (%)
S. cerevisiae (SK1 × Y12, CLR, 200X)	raw data	13988	87.80	1.91	7.32	2.97	93.4	37.7 / 36.6
	Canu	11606	99.53	0.11	0.16	0.20	89.8	94.3 / 95.9
	FALCON	10400	99.69	0.08	0.03	0.20	88.5	73.3 / 82.2
	MECAT2	11734	99.37	0.09	0.46	0.08	87.4	90.5 / 88.0
	PECAT	11785	99.71	0.02	0.12	0.15	99.7	98.7 / 99.2
A. thaliana (Col-0 × Cvi-0, CLR, 164X)	raw data	26088	88.24	2.06	5.54	4.16	86.6	40.3 / 41.0
	Canu	23105	99.31	0.14	0.16	0.38	94.8	97.6 / 98.2
	FALCON	20530	99.39	0.11	0.10	0.40	92.7	89.9 / 90.5
	MECAT2	23285	99.27	0.13	0.48	0.12	94.4	96.5 / 97.1
	PECAT	23414	99.44	0.15	0.13	0.28	99.7	98.9 / 99.4
D. melanogaster (ISO1 × A4, CLR, 200X)	raw data	60067	89.58	2.72	5.14	2.56	93.6	63.1 / 64.8
	Canu	54896	99.45	0.11	0.24	0.20	91.4	93.5 / 97.2
	FALCON	37210	98.90	0.05	0.28	0.76	93.4	90.7 / 93.8
	MECAT2	47894	99.01	0.08	0.77	0.14	90.0	91.5 / 94.6
	PECAT	51149	99.57	0.03	0.23	0.17	99.9	94.7 / 98.6
B. taurus (Angus × Brahman, CLR, 135X)	raw data	26938	86.25	3.09	7.02	3.64	87.7	12.9 / 12.1
	MECAT2	21517	98.92	0.12	0.80	0.17	85.3	88.5 / 88.9
	PECAT	21760	99.40	0.07	0.18	0.35	99.7	96.9 / 97.2
A. thaliana (Col-0 × C24, ONT, 106X)	raw data	30858	92.33	2.79	1.81	3.06	95.4	90.1 / 89.4
	Canu	30092	98.64	0.39	0.15	0.82	89.1	90.4 / 89.6
	NECAT	29551	98.95	0.32	0.29	0.45	92.4	88.8 / 88.0
	PECAT	29799	99.00	0.39	0.15	0.46	99.4	96.7 / 96.0
B. taurus (Bison × Simmental, ONT, 200X)	raw data	83396	89.12	3.58	2.68	4.62	93.7	69.4 / 69.4
	NECAT	82988	98.78	0.31	0.36	0.55	85.7	80.2 / 80.9
	PECAT	80587	98.61	0.26	0.20	0.92	99.3	92.2 / 93.5
HG002 (ONT, R9 59X)	raw data	107537	94.97	1.81	1.24	1.97	98.8	98.6 / 98.6
	NECAT	106400	99.35	0.35	0.17	0.12	91.1	74.8 / 64.8
	PECAT	104009	99.85	0.04	0.04	0.08	99.9	98.8 / 99.1

'Match', 'Mismatch', 'Insertion', and 'Deletion' are the percentages of matches, mismatches, insertions, and deletions in the alignments between the reads and the reference genome. 'Size' is the total number of base pairs of the corrected reads. 'N50' is the length of the shortest read for which longer and equal-length reads cover at least 50% of the 40X longest reads. 'Consistency' is defined as $\sum \max(k_p, k_m)/\sum(k_p + k_m)$, in which k_p and k_m are the number of paternal and maternal haplotype-specific k-mers in each read. 'Completeness' is the percentage of parent-specific k-mers (occurrences ≥ 4) in the 40X longest reads. 'PAT' and 'MAT' are paternal Completeness and maternal Completeness respectively.

Supplementary Table 7. Accuracy of raw reads and corrected reads by NECAT and PECAT in difficult-to-map regions and low-complexity regions of HG002 reference genome

Region	raw data	NECAT	PECAT
Segmental duplications	94.83%	98.90%	99.79%
Low-mappability regions	94.94%	98.77%	99.57%
250-bp+ non-unique regions	94.45%	98.63%	99.43%
Homopolymer (7-11 bp)	93.77%	98.97%	99.44%
Homopolymer (>11 bp)	93.27%	97.61%	98.54%
Dimer repeat regions (11-50bp)	94.39%	98.75%	99.52%
Trimer repeat regions (15-50 bp)	94.96%	99.21%	99.77%

Supplementary Table 8. Precision, recall, and F1-score of small variants (SNP, INDEL) and structural variants (SV) in HG002 assemblies.

Pipeline	SNP			INDEL			SV		
	Precision (%)	Recall (%)	F1-score (%)	Precision (%)	Recall (%)	F1-score (%)	Precision (%)	Recall (%)	F1-score (%)
Hifiasm (pri/alt)	99.8	92.4	96.0	97.9	90.4	94.0	92.6	98.1	95.3
Hifiasm (dual)	99.6	99.0	99.3	97.4	97.1	97.3	94.5	97.8	96.1
Flye+Hapdup (dual)	97.7	99.1	98.4	23.4	68.8	34.9	94.2	97.7	95.9
Shasta (pri/alt)	90.0	79.9	84.7	18.9	44.1	26.5	93.6	86.7	90.0
PECAT (pri/alt)	96.4	98.6	97.5	19.3	61.1	29.3	93.9	98.1	96.0
PECAT (dual)	96.4	98.5	97.4	19.3	61.1	29.3	94.2	98.0	96.0

The assemblies by Hifiasm are from HiFi reads. The assemblies by other methods are from Nanopore R9 reads. 'pri/alt' represents primary/alternate format. 'dual' represents dual assembly format.

Supplementary Table 9. Running time of error correction methods.

Dataset	Pipeline	Size (G)	Time (H)	Speed (G/H)
S. cerevisiae SK1×Y12, CLR, 200X	Canu	1.11	70	0.016
	MECAT2	1.72	6	0.300
	PECAT	0.75	7	0.111
A. thaliana Col-0 × Cvi-0, CLR, 164X	Canu	12.41	712	0.017
	MECAT2	17.89	134	0.133
	PECAT	8.30	90	0.093
D. melanogaster ISO1 × A4, CLR, 200X	Canu	13.30	1497	0.009
	MECAT2	23.12	223	0.104
	PECAT	9.28	98	0.095
B. taurus Angus × Brahman, CLR, 135X	MECAT2	232.05	5578	0.042
	PECAT	151.13	3254	0.046
A. thaliana Col-0 × C24, ONT, 106X	Canu	11.21	726	0.015
	NECAT	9.27	75	0.123
	PECAT	9.93	109	0.091
B. taurus Bison × Simmental, ONT, 200X	NECAT	217.00	13524	0.016
	PECAT	205.74	4217	0.049
HG002, ONT, R9, 59X	NECAT	171.99	5637	0.031
	PECAT	165.32	4404	0.038

'Size' is the total number of base pairs of the corrected reads. 'Time' is the running time of the error correction method, and the 'Speed' is defined as 'Size'/'Time'. All the pipelines are tested on the same computer with a 2.0 GHz CPU and 2T GB RAM of memory and run with 48 threads. For *B. taurus* and HG002, Canu didn't correct the raw reads in 3 weeks, so it is excluded. Since FALCON does not have an independent error correction step, the table doesn't contain its running times.

Supplementary Table 10. Running time, memory usage, and disk space usage of assemblers.

Dataset	Pipeline	CPU time (H)	Peak memory usage (G)	Peak disk space usage (G)
S. cerevisiae SK1×Y12, CLR, 200X	Canu	109	7	-
	FALCON-Unzip	199	7	-
	PECAT	11	18	4
A. thaliana Col-0×Cvi-0, CLR, 135X	Canu	1456	12	-
	FALCON-Unzip	2652	35	-
	PECAT	167	71	80
D. melanogaster ISO1×A4, CLR, 164X	Canu	3006	34	-
	FALCON-Unzip	3528	30	-
	PECAT	142	41	49
B. taurus Angus×Brahman, CLR, 135X	PECAT	4437	219	1099
A. thaliana Col-0×C24, ONT, 106X	Canu	8359	29	-
	Flye+Hapdup	202	94	-
	Shasta	9	60	-
	PECAT	359	179	142
B. taurus Bison×Simmental, ONT, 200X	Flye+Hapdup	8159	387	-
	Shasta	519	1357	-
	PECAT	8869	381	1574
HG002, ONT, R9, 59X	Flye+Hapdup	3020	430	-
	Shasta	153	781	-
	PECAT	7456	348	1211

All the pipelines are tested on the same computer with a 2.0 GHz CPU and 2T GB RAM of memory and run with 48 threads. Since FALCON-Unzip and Canu didn't generate the assemblies in 3 weeks, it is excluded on datasets *B. taurus* and HG002.

Supplementary Table 11. Performance comparison of error correction by PECAT on HG002 datasets using different sequencing techniques.

Dataset	Pipeline	N50	Match (%)	Mismatch (%)	Insertion (%)	Deletion (%)	Consistency (%)	Completeness PAT (%) / MAT (%)
ONT, R9, UL, 59X	raw data	107537	94.97	1.81	1.24	1.97	98.75	98.6 / 98.6
	PECAT	104009	99.85	0.04	0.04	0.08	99.85	98.8 / 99.1
ONT, R10, UL, 116X	raw data	235027	98.25	0.61	0.48	0.66	99.64	99.4 / 99.7
	PECAT	210995	99.88	0.02	0.03	0.06	99.98	99.3 / 99.7
ONT, R10, Duplex, 45X	raw data	37531	99.67	0.08	0.09	0.17	99.80	99.4 / 99.8
	PECAT	36780	99.96	0.00	0.01	0.03	99.96	99.2 / 99.6
HiFi, 36X	raw data	14718	99.77	0.01	0.10	0.11	99.87	99.4 / 99.8
	PECAT	14661	99.99	0.00	0.00	0.01	99.98	99.2 / 99.6

'Match', 'Mismatch', 'Insertion', and 'Deletion' are the percentages of matches, mismatches, insertions, and deletions in the alignments between the reads and the reference genome. 'Size' is the total number of base pairs of the corrected reads. 'N50' is the length of the shortest read for which longer and equal-length reads cover at least 50% of the 40X longest reads. 'Consistency' is defined as $\sum \max(k_p, k_m)/\sum(k_p + k_m)$, in which k_p and k_m are the number of paternal and maternal haplotype-specific k-mers in each read. 'Completeness' is the percentage of parent-specific k-mers (occurrences ≥ 4) in the 40X longest reads. 'PAT' and 'MAT' are paternal Completeness and maternal Completeness respectively. 'ONT' indicates the dataset is composed of Nanopore reads. 'UL' indicates the reads are ultra-long reads. 'Duplex' indicates the dataset is generated by the duplex sequencing method. 'HiFi' indicates the dataset is composed of PacBio HiFi reads.

Supplementary Table 12. Performance comparison of assembly on Nanopore R10 and PacBio HiFi reads.

Dataset	Pipeline	Size (Mb)	NG50 (Mb)	Quality (reference-based)	Quality (k-mer-based)	BUSCO (%)	Hamming error (%)	Phase block NG50 (Mb)	Intra-block switch error (%)
	Ref	2959.3/3061.7	146.7/154.4	-/-	58.6/59.4	92.8/95.8	0.15/0.08	90.4/106.7	0.02/0.03
HG002 ONT, R10, UL, 116X	Flye+HapDup (dual)	2954.7/2952.1	61.9/59.3	36.4/36.2	48.9/48.2	95.8/95.7	3.13/3.02	46.5/39.1	0.02/0.03
	Shasta (pri/alt)	3095.4/2729.9	45.3/33.8	34.3/34.4	42.1/48.7	95.7/92.2	3.05/0.86	17.2/15.4	0.30/0.44
	PECAT (pri/alt)	3159.7/2895.6	91.4/59.9	38.0/38.0	49.0/50.1	95.8/92.4	3.75/0.30	59.4/58.0	0.04/0.05
	PECAT (dual)	3153.4/2917.8	91.4/80.2	38.0/38.0	49.2/50.0	95.8/92.7	2.99/1.49	63.8/59.2	0.04/0.06
HG002 ONT, R10, Duplex, 45X	Flye+HapDup (dual)	2921.2/2921.7	26.1/26.1	37.2/37.2	52.2/51.9	95.7/95.8	20.61/20.90	4.2/4.2	0.04/0.04
	Shasta (pri/alt)	3116.3/2292.6	38.6/0.8	36.8/36.0	48.6/57.0	95.4/75.3	21.11/0.31	2.1/0.7	0.28/0.23
	PECAT (pri/alt)	3035.8/2762.6	75.6/1.1	38.5/38.3	55.1/54.3	95.8/86.7	24.55/0.42	2.6/0.9	0.07/0.08
	PECAT (dual)	3029.0/2828.0	71.2/70.8	38.5/38.5	55.5/55.9	95.6/92.6	22.48/34.47	4.3/3.7	0.07/0.10
HG002 HiFi, 36X	Flye+HapDup (dual)	3211.4/3210.9	4.6/4.6	47.0/45.2	53.4/52.8	94.7/94.7	14.88/14.93	0.7/0.7	0.21/0.22
	Hifiasm (pri/alt) *	3112.2/2910.3	89.9/0.4	50.0/50.0	55.3/56.5	95.6/77.0	24.70/0.37	1.1/0.3	0.11/0.01
	Hifiasm (dual) *	3015.3/3077.5	44.8/64.5	50.0/50.0	54.9/57.8	95.5/94.9	34.80/24.10	1.0/1.0	0.16/0.11
	PECAT (pri/alt)	2991.8/2344.4	50.9/0.2	45.2/46.0	53.3/52.3	95.6/67.4	25.21/0.32	0.7/0.1	0.19/0.07
	PECAT (dual)	2990.8/2798.7	41.0/39.7	45.2/45.2	53.4/57.1	95.6/92.3	24.02/35.49	0.9/0.7	0.19/0.27

'Size' is the total number of base pairs in all contigs generated by assemblers. 'NG50' is the length of the shortest contig for which longer and equal length contigs cover at least 50 of genome size. The genome sizes of HG002 that we used for evaluation is 3G. 'BUSCO' is gene completeness evaluated by BUSCO. 'Hamming error' is the fraction of nondominant parental-specific k-mers in a contig. 'Quality (reference-based)' is the metric 'q50' evaluated by Pomoxis. 'Quality (k-mer-based)', 'Phase block NG50', and 'Intra-block switch error' are evaluated by mercury. 'pri/alt' represents primary/alternate assembly format. 'dual' represents dual assembly format. 'ONT' indicates the dataset is composed of Nanopore reads. 'UL' indicates the reads are ultra-long reads. 'Duplex' indicates the dataset is generated by the duplex sequencing method. 'HiFi' indicates the dataset is composed of PacBio HiFi reads. The two sets of contigs are separately reported in each cell. 'Ref' is the reference genome. The sources of the reference genomes are illustrated in **Supplementary Table 17**. Asterisks mark previously published assemblies.

Supplementary Table 13. Precision, recall, and F1-score of small variants (SNP, INDEL) and structural variants (SV) in HG002 assemblies from Nanopore R10 (ultra-long) reads.

Pipeline	SNP			INDEL			SV		
	Precision (%)	Recall (%)	F1-score (%)	Precision (%)	Recall (%)	F1-score (%)	Precision (%)	Recall (%)	F1-score (%)
Flye+Hapdup (dual)	99.6	99.5	99.6	64.5	84.3	73.1	94.7	97.8	96.2
Shasta (pri/alt)	99.4	98.7	99.0	63.0	72.5	67.4	94.9	94.4	94.7
PECAT (pri/alt)	99.5	99.3	99.4	71.6	87.6	78.8	94.7	98.0	96.3
PECAT (dual)	99.5	99.5	99.5	71.6	87.8	78.9	94.7	98.0	96.3

‘pri/alt’ represents primary/alternate format. ‘dual’ represents dual assembly format.

Supplementary Table 14. Precision, recall, and F1-score of small variants (SNP, INDEL) and structural variants (SV) in HG002 assemblies from Nanopore R10 duplex reads.

Pipeline	SNP			INDEL			SV		
	Precision (%)	Recall (%)	F1-score (%)	Precision (%)	Recall (%)	F1-score (%)	Precision (%)	Recall (%)	F1-score (%)
Flye+Hapdup (dual)	99.7	99.5	99.6	64.9	85.3	73.8	94.6	97.9	96.2
Shasta (pri/alt)	99.7	89.7	94.4	70.1	66.0	68.0	94.8	96.2	95.5
PECAT (pri/alt)	99.6	96.9	98.2	74.5	85.9	79.8	93.7	97.9	95.8
PECAT (dual)	99.6	99.3	99.4	74.3	88.5	80.8	94.5	97.9	96.2

'pri/alt' represents primary/alternate format. 'dual' represents dual assembly format.

Supplementary Table 15. Precision, recall, and F1-score of small variants (SNP, INDEL) and structural variants (SV) in HG002 assemblies from HiFi reads.

Pipeline	SNP			INDEL			SV		
	Precision (%)	Recall (%)	F1-score (%)	Precision (%)	Recall (%)	F1-score (%)	Precision (%)	Recall (%)	F1-score (%)
Flye+Hapdup (dual)	99.4	95.6	97.5	96.3	95.7	96.0	94.5	93.4	94.0
Hifiasm (pri/alt)	99.8	92.4	96.0	97.9	90.4	94.0	92.6	98.1	95.3
Hifiasm (dual)	99.6	99.0	99.3	97.4	97.1	97.3	94.5	97.8	96.1
PECAT (pri/alt)	99.7	85.0	91.8	96.2	82.7	88.9	93.8	97.5	95.6
PECAT (dual)	99.5	98.7	99.1	96.5	96.8	96.6	94.8	97.5	96.1

'pri/alt' represents primary/alternate format. 'dual' represents dual assembly format.

Supplementary Table 16. Performance of assemblies by PECAT on HG002 Nanopore datasets with different coverage.

Dataset	Format	Size (Mb)	NG50 (Mb)	Quality (reference-based)	Quality (k-mer-based)	BUSCO (%)	Hamming error (%)	Phase block NG50 (Mb)	Intra-block switch error (%)
Ref	-	2959.3/3061.7	146.7/154.4	-/-	58.6/59.4	92.8/95.8	0.15/0.08	90.4/106.7	0.02/0.03
HG002 ONT, 37X	pri/alt	3061.4/2772.9	74.8/7.7	30.3/30.3	39.4/39.3	94.1/89.5	18.84/0.89	11.8/6.7	0.09/0.12
	dual	3055.7/2829.5	60.5/59.3	30.3/30.4	39.4/40.1	94.0/91.1	12.05/15.43	17.7/17.1	0.09/0.13
HG002 ONT, 59X	pri/alt	3059.3/2857.7	92.9/15.0	30.8/30.8	41.8/41.6	94.7/91.0	15.72/1.48	22.2/13.0	0.08/0.11
	dual	3057.2/2927.2	92.7/74.5	30.8/30.8	41.8/41.8	94.6/91.6	9.67/10.98	30.8/23.6	0.08/0.11

'Size' is the total number of base pairs in all contigs generated by assemblers. 'NG50' is the length of the shortest contig for which longer and equal length contigs cover at least 50 of genome size. The genome size of HG002 that we used for evaluation is 3G, respectively. 'BUSCO' is gene completeness evaluated by BUSCO. 'Quality (reference-based)' is the metric 'q50' evaluated by Pomoxis. 'Hamming error' is the fraction of nondominant parental-specific k-mers in a contig. 'Quality (k-mer-based)', 'Phase block NG50', and 'Intra-block switch error' are evaluated by mercury. 'pri/alt' represents primary/alternate assembly format. 'dual' represents dual assembly format. The two sets of contigs are separately reported in each cell. 'Ref' is the reference genome. The sources of the reference genomes are illustrated in **Supplementary Table 17**.

Supplementary Table 17. Detailed information of the datasets used in this study.

Dataset	Type	Data resource
NCTC9024<br>NCTC9006	PacBio CLR	SAMEA3340626, SAMEA3376915
S. cerevisiae<br>SK1×Y12	PacBio CLR	ERR1080522, ERR1080529, ERR1080536, ERR1080537, ERR1124245, ERR1140978 (SK1)<br>ERR1080526, ERR1080538, ERR1080539, ERR1140975, ERR1140979, ERR985361 (Y12)
	NGS	SRR4074258(SK1)<br>SRR4074358(Y12)
	Reference	ASM205788v1 (SK1)<br>ASM205864v1 (Y12)
A. thaliana<br>Col-0×Cvi-0	PacBio CLR	SRR3405242 - SRR3405290 (Col-0)<br>SRR3405327 - SRR3405386 (Cvi-0)<br>SRR3405291 - SRR3405326 (F1)
	NGS	SRR3703081, SRR3703082, SRR3703105 (F1)<br>SRR16841689 (Col-0)<br>ERR3624578 (Cvi-0)
	Reference	GCA_020911765.1 (Col-0)<br>GCA_902460275.1 (Cvi-0)
D. melanogaster<br>ISO1×A4	PacBio CLR	SRR9969843 (F1)
	NGS	SRR6702604 (ISO1)<br>SRR457665, SRR457666, SRR457707 (A4)
	Reference	GCA_000001215.4 (ISO1)<br>ASM340174v1 (A4)
B. taurus<br>Angus ×<br>Brahman	PacBio CLR	SRR6691718, SRR6691728 - SRR6691879, SRR6691882 -SRR6691900, SRR6691904, SRR6691905, SRR6691908 -SRR6691950, SRR6691954 - SRR6691960, SRR6691962 - SRR6691984 (F1)
	NGS	SRR6691901 - SRR6691903, SRR6691907 (Angus)<br>SRR6691719, SRR6691880, SRR6691881, SRR6691906 (Brahman)<br>SRR6691720 - SRR6691727, SRR6691748, SRR6691951- SRR6691953, SRR6691961 (F1)
	Reference	GCA_003369685.2 (Angus)<br>GCA_003369695.2 (Brahman)
A. thaliana<br>Col-0×C24	Nanopore	CRA008108 (F1) *
	NGS	SRR16841689 (Col-0)<br>ERR3624577 (C24)
	Reference	GCA_020911765.1 (Col-0)<br>GCA_902705455.1 (C24)
B. taurus<br>Bison ×<br>Simmental	Nanopore	SRR13105460 - SRR13105478 (F1)
	NGS	SRR13081814 (Bison)<br>SRR13081923 (Simmental)<br>SRR13081717 (F1)

	Reference	GCA_018282365.1 (Bison)<br>GCA_018282465.1 (Simmental)
HG002	Nanopore R9	https://s3-us-west-2.amazonaws.com/human-pangenomics/index.html?prefix=T2T/scratch/HG002/sequencing/ont/<br>03_08_22_R941_HG002_1_Guppy_6.0.6_prom_sup.fastq.gz<br>03_08_22_R941_HG002_2_Guppy_6.0.6_prom_sup.fastq.gz
	Nanopore R10	https://s3-us-west-2.amazonaws.com/human-pangenomics/index.html?prefix=submissions/5b73fa0e-658a-4248-b2b8-cd16155bc157--UCSC_GIAB_R1041_nanopore/HG002_R1041_UL/Guppy6/
	Nanopore R10 Duplex	https://s3-us-west-2.amazonaws.com/human-pangenomics/index.html?prefix=submissions/0CB931D5-AE0C-4187-8BD8-B3A9C9BFDADE--UCSC_HG002_R1041_Duplex_Dorado/Dorado_v0.1.1/stereo_duplex
	PacBio HiFi	SRR10382244, SRR10382245, SRR10382248, SRR10382249
	NGS	https://s3-us-west-2.amazonaws.com/human-pangenomics/index.html?prefix=NHGRI_UCSC_panel/HG002/hpp_HG002_NA24385_son_v1/
	Reference	GCA_021950905.1 (HG003)<br>GCA_021951015.1 (HG004)

NGS: Next-generation sequencing. The datasets marked with asterisks are available at https://ngdc.cncb.ac.cn/. The other datasets are available at https://www.ncbi.nlm.nih.gov/.

Supplementary References

1. Nurk, S. *et al.* HiCanu: accurate assembly of segmental duplications, satellites, and allelic variants from high-fidelity long reads. *Genome Res.* **30**, 1291–1305 (2020).
2. Zhang, Q. *et al.* The chromatin remodeler DDM1 promotes hybrid vigor by regulating salicylic acid metabolism. *Cell Discov.* **2**, 16027 (2016).
3. Ono, Y., Asai, K. & Hamada, M. PBSIM2: a simulator for long-read sequencers with a novel generative model of quality scores. *Bioinformatics* **37**, 589–595 (2021).
4. Koren, S. *et al.* Canu: scalable and accurate long-read assembly via adaptive *k* -mer weighting and repeat separation. *Genome Res.* **27**, 722–736 (2017).
5. Guan, D. *et al.* Identifying and removing haplotypic duplication in primary genome assemblies. *Bioinformatics* **36**, 2896–2898 (2020).
6. Chin, C.-S. *et al.* Phased diploid genome assembly with single-molecule real-time sequencing. *Nat. Methods* **13**, 1050–1054 (2016).
7. Xiao, C.-L. *et al.* MECAT: fast mapping, error correction, and de novo assembly for single-molecule sequencing reads. *Nat. Methods* **14**, 1072–1074 (2017).
8. Chen, Y. *et al.* Efficient assembly of nanopore reads via highly accurate and intact error correction. *Nat. Commun.* **12**, 60 (2021).
9. Kolmogorov, M., Yuan, J., Lin, Y. & Pevzner, P. A. Assembly of long, error-prone reads using repeat graphs. *Nat. Biotechnol.* **37**, 540–546 (2019).
10. Shafin, K. *et al.* Haplotype-aware variant calling with PEPPER-Margin-DeepVariant enables high accuracy in nanopore long-reads. *Nat. Methods* **18**, 1322–1332 (2021).

11. Kolmogorov, M. *et al. Scalable Nanopore sequencing of human genomes provides a comprehensive view of haplotype-resolved variation and methylation*. http://biorxiv.org/lookup/doi/10.1101/2023.01.12.523790 (2023) doi:10.1101/2023.01.12.523790.

12. Shafin, K. *et al.* Nanopore sequencing and the Shasta toolkit enable efficient de novo assembly of eleven human genomes. *Nat. Biotechnol.* **38**, 1044–1053 (2020).

13. Ranallo-Benavidez, T. R., Jaron, K. S. & Schatz, M. C. GenomeScope 2.0 and Smudgeplot for reference-free profiling of polyploid genomes. *Nat. Commun.* **11**, 1432 (2020).

14. Marçais, G. & Kingsford, C. A fast, lock-free approach for efficient parallel counting of occurrences of k-mers. *Bioinformatics* **27**, 764–770 (2011).

15. Manni, M., Berkeley, M. R., Seppey, M., Simão, F. A. & Zdobnov, E. M. BUSCO Update: Novel and Streamlined Workflows along with Broader and Deeper Phylogenetic Coverage for Scoring of Eukaryotic, Prokaryotic, and Viral Genomes. *Mol. Biol. Evol.* **38**, 4647–4654 (2021).

16. Gurevich, A., Saveliev, V., Vyahhi, N. & Tesler, G. QUAST: quality assessment tool for genome assemblies. *Bioinformatics* **29**, 1072–1075 (2013).

17. Li, H. *et al.* A synthetic-diploid benchmark for accurate variant-calling evaluation. *Nat. Methods* **15**, 595–597 (2018).

18. the Global Alliance for Genomics and Health Benchmarking Team *et al.* Best practices for benchmarking germline small-variant calls in human genomes. *Nat. Biotechnol.* **37**, 555–560 (2019).

19. English, A. C., Menon, V. K., Gibbs, R. A., Metcalf, G. A. & Sedlazeck, F. J. Truvari: refined structural variant comparison preserves allelic diversity. *Genome Biol.* **23**, 271 (2022).

20. Zook, J. M. *et al.* A robust benchmark for detection of germline large deletions and insertions. *Nat. Biotechnol.* (2020) doi:10.1038/s41587-020-0538-8.
