# Supplementary material for: De novo diploid genome assembly using long noisy reads
Source: Nat Commun. 2024 Apr 5;15:2964. doi: 10.1038/s41467-024-47349-7 (PMC10997618; doi:10.1038/s41467-024-47349-7)
Supplement: Supplementary file 3 — Description of Additional Supplementary Files [file 41467_2024_47349_MOESM3_ESM.docx]

**Description of additional supplementary files**

File Name: **Supplementary Data 1**

Description: QUAST statistics for genome assemblies
