# Supplementary material for: De novo diploid genome assembly using long noisy reads
Source: Nat Commun. 2024 Apr 5;15:2964. doi: 10.1038/s41467-024-47349-7 (PMC10997618; doi:10.1038/s41467-024-47349-7)
Supplement: Supplementary file 5 — Reporting Summary [file 41467_2024_47349_MOESM5_ESM.pdf]

nature portfolio

Corresponding author(s): Chuanle Xiao, Feng Luo, Jianxin Wang

Last updated by author(s): Mar 9, 2024

Reporting Summary

Nature Portfolio wishes to improve the reproducibility of the work that we publish. This form provides structure for consistency and transparency in reporting. For further information on Nature Portfolio policies, see our Editorial Policies and the Editorial Policy Checklist.

Statistics

For all statistical analyses, confirm that the following items are present in the figure legend, table legend, main text, or Methods section.

n/a	Confirmed	
☐	☒	The exact sample size (*n*) for each experimental group/condition, given as a discrete number and unit of measurement
☐	☒	A statement on whether measurements were taken from distinct samples or whether the same sample was measured repeatedly
☒	☐	The statistical test(s) used AND whether they are one- or two-sided<br>*Only common tests should be described solely by name; describe more complex techniques in the Methods section.*
☒	☐	A description of all covariates tested
☒	☐	A description of any assumptions or corrections, such as tests of normality and adjustment for multiple comparisons
☒	☐	A full description of the statistical parameters including central tendency (e.g. means) or other basic estimates (e.g. regression coefficient) AND variation (e.g. standard deviation) or associated estimates of uncertainty (e.g. confidence intervals)
☒	☐	For null hypothesis testing, the test statistic (e.g. *F*, *t*, *r*) with confidence intervals, effect sizes, degrees of freedom and *P* value noted<br>*Give P values as exact values whenever suitable.*
☒	☐	For Bayesian analysis, information on the choice of priors and Markov chain Monte Carlo settings
☒	☐	For hierarchical and complex designs, identification of the appropriate level for tests and full reporting of outcomes
☒	☐	Estimates of effect sizes (e.g. Cohen's *d*, Pearson's *r*), indicating how they were calculated

Our web collection on statistics for biologists contains articles on many of the points above.

Software and code

Policy information about availability of computer code

Data collection	No software was used for data collection. All datasets are available at public websites.
Data analysis	This study utilized open software and our shell scripts described in the supplementary note. The software introduced in this study is PECAT (v0.0.3)[https://github.com/lemene/PECAT]. Other softwares are Canu (v2.1), purge_dups (v1.2.5), FALCON (1.8.1), FALCON-Unzip (1.3.7), MECAT2 (f54c542), NECAT (47c6c23), Flye (2.9), Hapdup (0.5), Shasta (0.9.0), , Guppy(v5.0.16), merqury(v1.1), BUSCO (5.2.2), minimap2(2.22) and racon(v1.4.3).

For manuscripts utilizing custom algorithms or software that are central to the research but not yet described in published literature, software must be made available to editors and reviewers. We strongly encourage code deposition in a community repository (e.g. GitHub). See the Nature Portfolio guidelines for submitting code & software for further information.

Data

Policy information about availability of data

All manuscripts must include a data availability statement. This statement should provide the following information, where applicable:

- Accession codes, unique identifiers, or web links for publicly available datasets
- A description of any restrictions on data availability
- For clinical datasets or third party data, please ensure that the statement adheres to our policy

All described datasets are obtained from public websites, except for A. thaliana (Col-0 × C24) which is generated using our in-house sequencing. It is available from

NGDC at PRJCA011723 [https://ngdc.cncb.ac.cn/bioproject/browse/PRJCA011723]. S. cerevisiae (SK × Y12), A. thaliana (Col-0 × Cvi-0), D. melanogaster (ISO1 × A4), B. taurus (Angus × Brahman), and B. taurus (Bison×Simmental) are available from NCBI at PRJEB7245 [https://www.ncbi.nlm.nih.gov/bioproject/PRJEB7245], PRJNA314706 [https://www.ncbi.nlm.nih.gov/bioproject/PRJNA314706], PRJNA558397 [https://www.ncbi.nlm.nih.gov/bioproject/PRJNA558397], PRJNA432857 [https://www.ncbi.nlm.nih.gov/bioproject/PRJNA432857], and PRJNA677946 [https://www.ncbi.nlm.nih.gov/bioproject/PRJNA677946]. HG002 using Nanopore R9 sequencing is available at Human Pangenome Reference Consortium (HPRC) [https://s3-us-west-2.amazonaws.com/human-pangenomics/index.html?prefix=T2T/scratch/HG002/sequencing/ont/]. HG002 using Nanopore R10 sequencing is available at HPRC [https://s3-us-west-2.amazonaws.com/human-pangenomics/index.html?prefix=submissions/5b73fa0e-658a-4248-b2b8-cd16155bc157--UCSC_GIAB_R1041_nanopore/HG002_R1041_UL/Guppy6/]. HG002 using Nanopore R10 duplex sequencing is available at HPRC [https://s3-us-west-2.amazonaws.com/human-pangenomics/index.html?prefix=submissions/0CB931D5-AE0C-4187-8BD8-B3A9C9BFDADE--UCSC_HG002_R1041_Duplex_Dorado/Dorado_v0.1.1/stereo_duplex]. HG002 using PacBio HiFi sequencing is available from NCBI at PRJNA586863 [https://www.ncbi.nlm.nih.gov/bioproject/PRJNA586863]. GIAB v2.0 genome stratification BED files are available at GIAB [https://ftp-trace.ncbi.nlm.nih.gov/ReferenceSamples/giab/release/genome-stratifications/v2.0/GRCh38/]. HG002 GIAB benchmark is available at GIAB [https://ftp-trace.ncbi.nlm.nih.gov/giab/ftp/release/AshkenazimTrio/HG002_NA24385_son/NISTv3.3.2/]. The curated set of SVs in the HG002 is available at GIAB [https://ftp-trace.ncbi.nlm.nih.gov/giab/ftp/data/AshkenazimTrio/analysis/NIST_SVs_Integration_v0.6/]. NCTC9006 and NCTC9024 are available from ENA at PRJEB6403 [https://www.ebi.ac.uk/ena/browser/view/PRJEB6403]. The details of the datasets used in this study are reported in Supplementary Note 1 and Supplementary Table 17. All assemblies are available at Zenodo [https://doi.org/10.5281/zenodo.10457427].

Research involving human participants, their data, or biological material

Policy information about studies with human participants or human data. See also policy information about sex, gender (identity/presentation), and sexual orientation and race, ethnicity and racism.

Reporting on sex and gender	Not applicable since the method proposed by the manuscript is an error correction and assembly method that does not involve human research participants.
Reporting on race, ethnicity, or other socially relevant groupings	Not applicable since the method proposed by the manuscript is an error correction and assembly method that does not involve human research participants.
Population characteristics	Not applicable since the method proposed by the manuscript is an error correction and assembly method that does not involve human research participants.
Recruitment	Not applicable since the method proposed by the manuscript is an error correction and assembly method that does not involve human research participants.
Ethics oversight	Not applicable since the method proposed by the manuscript is an error correction and assembly method that does not involve human research participants.

Note that full information on the approval of the study protocol must also be provided in the manuscript.

Field-specific reporting

Please select the one below that is the best fit for your research. If you are not sure, read the appropriate sections before making your selection.

☒ Life sciences ☐ Behavioural & social sciences ☐ Ecological, evolutionary & environmental sciences

For a reference copy of the document with all sections, see nature.com/documents/nr-reporting-summary-flat.pdf

Life sciences study design

All studies must disclose on these points even when the disclosure is negative.

Sample size	Not applicable since no statistical tests were performed. The methods introduced by this study is deterministic. However, samples with available data no less 40x coverage are recommended for better assembly performance.
Data exclusions	No data were excluded from analysis
Replication	Not applicable since this study introduces deterministic methods which does not involve statistic analysis. We used multiple datasets to test robustness
Randomization	Not applicable since this study introduces deterministic methods. The datasets used by the methods are not randomly downsampled.
Blinding	Not applicable since this study does not involve statistic analysis. The methods used all the data to assemble the genomes.

Reporting for specific materials, systems and methods

We require information from authors about some types of materials, experimental systems and methods used in many studies. Here, indicate whether each material, system or method listed is relevant to your study. If you are not sure if a list item applies to your research, read the appropriate section before selecting a response.

Materials & experimental systems

n/a	Involved in the study
☒	☐ Antibodies
☒	☐ Eukaryotic cell lines
☒	☐ Palaeontology and archaeology
☒	☐ Animals and other organisms
☒	☐ Clinical data
☒	☐ Dual use research of concern
☐	☒ Plants

Methods

n/a	Involved in the study
☒	☐ ChIP-seq
☒	☐ Flow cytometry
☒	☐ MRI-based neuroimaging

Plants

Seed stocks	Seeds of Arabidopsis thaliana ecotypes Col-0 (SN:SN33291) and C24 (SN:WT0025) were obtained from AraShare (https://www.arashare.cn).
Novel plant genotypes	The sequenced hybrid seedling (Col-0 × C24) was acquired by crossing of Col-0 and C24
Authentication	Not available
